# Geography of Life Histories in a Tropical Fauna: The Case of Indian Butterflies

**DOI:** 10.1002/ece3.72217

**Published:** 2025-09-22

**Authors:** Gaurab Nandi Das, Zdenek Faltynek Fric, Martin Konvicka

**Affiliations:** ^1^ Institute of Entomology Biology Centre CAS Ceske Budejovice Czech Republic; ^2^ Faculty of Science University of South Bohemia Ceske Budejovice Czech Republic; ^3^ Faculty of Environmental Sciences Czech University of Life Sciences Prague Prague Czech Republic

**Keywords:** faunal structure, functional traits, Lepidoptera, oriental realm, South Asia

## Abstract

The butterfly fauna of the megadiverse Republic of India contains 1386 species. The species richness in its 36 federal states and union territories primarily reflects the peculiar geography of the Indian peninsula, which remains unexplored except for patterns influencing species richness. Here, we develop on previous findings by focusing on species identities, by exploring the impact of physical geography, climate, land covers, socioeconomic conditions of the states, patterns by life history traits and shared evolutionary history of butterflies on the presence of individual species per federal state across the Indian peninsula. Physical geography was the strongest predictor of the states' butterfly fauna compositions, followed by climate, land covers and socioeconomics. The main faunal structures separate the humid Northeast from the rest of the country; distinguish humid Western Ghats states from the rest; and group together peninsular mountains. Analysing life histories showed that butterflies of the humid northeastern and southwestern states tend to be larger and develop on woody plants or large grasses; those of arid and high‐altitude states tend to be smaller and develop on small forbs; whereas those utilising broad larval host plant scopes tend to be associated with shrubs and vines and inhabit large geographic ranges. More information on Indian butterflies' life histories would likely yield more intricate insights.

## Graphical abstract

The current study examines the butterfly species composition across India's 36 states and territories by utilising an updated checklist and ordination analyses. It incorporates life history traits to provide a deeper ecological understanding. Although India's butterfly fauna has been extensively researched, analyses based on trait composition are a novel approach.

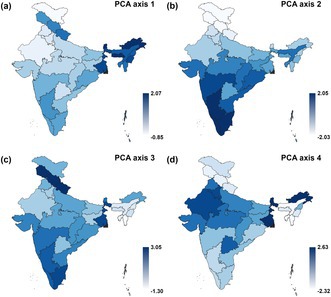

## 1 Introduction

Fauna of land masses reflect geological history, biotic evolution including speciation, dispersal and extinction and life‐supporting conditions, such as available energy/climate, which determine the biomes/landscape types and vegetation, and ultimately, resources for the animal components of ecosystems (e.g., Williams et al. 2015; Somveille et al. 2018; Rosenberg et al. 2023). The accelerating transformation of ecosystems and novel interactions peculiar to the current Anthropocene epoch (Malhi 2017) represent yet another level of factors influencing the distribution of species.

The monophyletic clade of butterflies (Lepidoptera: Papilionoidea) might represent a richer source of insights into the diversity‐distribution dynamics than birds, perhaps the most popular group in biogeography and macroecology (e.g., Pigot et al. 2018; Storch et al. 2023). Butterflies are similarly species‐rich and easy to record. They are ecologically more homogeneous than birds, although their role in ecosystems is rarely verbalised. While their larvae convey to a general image of diet‐specialised insect herbivores (Janz et al. 2001; Clarke 2022), their adults feed on high‐sugar liquids, including nectar, honeydew, sap and rotting fruit. As rather inefficient pollinators (Wiklund et al. 1979; Bauder et al. 2015; but see Burgin et al. 2023), they exploit plants × pollinators or Hemiptera × ants' interactions (Corke 1999; Gardner‐Gee et al. 2014). As interaction exploiters, butterflies' diversity should follow the complexity of interactions, which is highest in the humid tropics (Trøjelsgaard and Olesen 2013; Forister et al. 2014). The butterfly clade originated in the tropics of the Americas (Kawahara et al. 2023) and displays the highest alpha‐diversity in humid tropical forests (Brown and Freitas 2000; Bonebrake et al. 2010). From them, these interaction exploiters colonised less complex ecosystems.

Yet, detailed knowledge of butterfly faunas and communities exists mainly for those regions of Earth where lepidopterology as a hobby (Salmon et al. 2000) and studies of butterflies as an academic niche (Kudrna 1990) historically evolved: Europe, parts of North America (Scott 1986; Settele et al. 2009), less so Australia, Japan, or South Africa (Braby 2000; Mecenero et al. 2013; Japan Butterfly Conservation Association 2019). This selection includes both regions with long cultural continuity (Europe, Japan) and regions that underwent major ethnocultural transformations (Australia, North America). It is rather uniform, however, in terms of background ecological conditions, as they are mainly situated in temperate bioclimatic zones. From these regions, near‐complete knowledge of life histories of entire butterfly faunas exists (e.g., Baguette and Stevens 2013; Middleton‐Welling et al. 2020; Seifert and Fiedler 2024). The knowledge of life histories, formalised as life history traits' analysis (McGill et al. 2006; de Bello et al. 2021), is instrumental for understanding mechanisms of assemblages' composition and underlying ecosystem processes (Diamond et al. 2011; Slancarova et al. 2016; Wong et al. 2019).

The Republic of India is a megadiverse, prevailingly tropical country with a venerable tradition of butterfly research (Evans 1932; Holloway 1974; Mani 1986), a recent boom in butterfly distribution and ecology studies (e.g., Dar et al. 2022; Dewan et al. 2022; Jambhekar and Driscoll 2023; Naik et al. 2022), and over 1300 butterfly species recorded to date (Das et al. 2023). Belonging to the Indo‐Malayan zoogeography realm, the country displays a complex geography with the World's highest mountains separating it from the Palearctic Realm in the North, southern mountain ranges acting as regional endemism centres, an ocean separating it from other tropical regions, and only narrow conduits towards the rest of the Indo‐Malayan realm in the East and to arid parts of the Palearctic realm in the West. Analysing the species richness of 36 federal states and territories, Das et al. (2023) showed that due to peculiarities of Indian geography, some of the well‐established macroecology patterns, such as the species‐area relationship or latitudinal decrease of species richness, do not hold for this system, whereas topographic diversity and the precipitation/temperature ratio (energy availability) displayed strong effects. As found earlier (Holloway 1974; Kunte 2016; Dewan et al. 2022), humid and forested northeastern states were the species richest, mountainous southwest harboured the highest endemism, and both the core of the peninsula with a more continental climate and the arid northwest were species‐poor.

Species richness, however, is a simplistic description of regional ecological conditions. Exploring the species' composition of regional faunas should bring deeper insights into factors structuring the faunas, especially if studied through the prism of historical traits (Dennis et al. 1998; Kleisner et al. 2012; Soininen et al. 2016). The latter are not yet available for Indian butterflies in the detail typically analysed from temperate regions (Middleton‐Welling et al. 2020), but some life history knowledge is available for all Indian species (cf. Kehimkar 2008; Shirey et al. 2022; Savela 2023), with more detailed knowledge existing for regional faunas (Dewan et al. 2022). Additionally, the patterns of distribution of the majority of species are known at a global level.

Here, we capitalise on the existing knowledge of distribution and life histories of Indian butterflies and use multivariate techniques to disclose the patterns of species composition at the level of federal states and territories. We relate the patterns to predictors characterising the states, namely geography, climate, land covers and current socioeconomic conditions. We then interpret the patterns found by available species traits. Because species ranges and life histories are closely tied to phylogeny, we use phylogeny‐controlled analysis to differentiate between phylogeny‐linked and unlinked patterns.

We target the following hypotheses: (1) Unconstrained ordinations will reveal faunal structures (*sensu* Dennis et al. 1998) in species co‐occurrence, defined as similarities or dissimilarities among states and regions. (2) These faunal structures should be best interpretable by physical geography, which reflects long‐lasting Earth‐forming processes, and by climate, reflecting energy and resource availability. (3) Measurements of land covers provide a robust but imperfect information on ecosystems composition, while socioeconomic conditions mainly reflect recent processes in human society; socioeconomic predictors should only weakly predict the species compositions. Regarding life history traits, (4) butterfly body size should increase with energy availability and climatic stability. (5) The growth forms of larval host plants should reflect the representation of plant growth forms in the respective biomes. (6) Species with wide global distribution should develop on wider scopes of host plant forms than species utilising narrow scopes of host plant forms.

## 2 Materials and Methods

### 2.1 Data Preparation

We used the matrix of all Indian butterfly species and their presences in 36 federal states and union territories (Das et al. 2023; Figure S1) and updated it with more recent records, obtaining a total of 1386 species by April 2024.

Predictors describing individual states (Table 1) developed on those used by Das et al. (2023) in the study of species richness patterns. The six *geography* predictors were the cardinal coordinates of the states' centroids, their average altitude, their areas, and the difference between highest and lowest altitude viewed as a measure of topographic and habitat heterogeneity. For *climate*, we used the 19 bioclimatic variables (WorldClim 2024), from CHELSA v.1.2 with 2.5 min spatial resolution (Karger et al. 2017), extracted and averaged for the areas within the federal states' boundaries. For *land covers*, we used 24 statistics describing the conditions in Indian federal states, extracted using satellite imagery and provided by Bhuvan LULC 15–16 (Bhuvan 2023). The six *socioeconomic* variables (population density, urban population, rural population, literacy rate, gross domestic product per capita and total livestock) were publicly available statistics describing the conditions of individual states, extracted from Das et al. (2023).

**TABLE 1:** Overview of the predictors used to analyse the distribution of 1386 butterfly species recorded in 36 federal states and union territories of the Republic of India. The predictors marked as * and ** are compound predictors, obtained by PCA analyses of *Climate* and *Land covers* predictors, respectively (see Figures S2 and S3 for details).

Variables	Description	Unit	Variables	Description	Unit
**Geography**			**Land covers**		
*x*	Centroid latitude	DD	Cr	Crop land	sq. km.
*y*	Centroid longitude	DD	Sh	Current shifting cultivation	sq. km.
*z*_min_	Minimum altitude	m	Fa	Fallow	sq. km.
*z*_max_	Maximum altitude	m	Pl	Plantation	sq. km.
*z*	Mean altitude	m	Ba	Barren rocky	sq. km.
Area	Area of the state	sq. km.	Gu	Gullied/Ravinous	sq. km.
AltDiff	Altitude difference (*z*_max_ – *z*_min_)	m	Ra	Rann	sq. km.
**Climate**			Sa	Salt affected land	sq. km.
bio1	Annual mean temperature	SD	Sandy area	sq. km.	
bio2	Mean diurnal range (mean of monthly (max temp – min temp))		ScL	Scrub land	sq. km.
			Mi	Mining	sq. km.
bio3	Isothermality (bio2/bio7) (×100)		Ru	Rural	sq. km.
bio4	Temperature aeasonality (standard deviation ×100)	°C	Ur	Urban	sq. km.
			De	Deciduous	sq. km.
bio5	Max temperature of warmest month	°C	Ev	Evergreen/Semi Evergreen	sq. km.
bio6	Min temperature of coldest month	°C	FoP	Forest plantation	sq. km.
bio7	Temperature annual range (bio5‐bio6)	°C	ScF	Scrub forest	sq. km.
bio8	Mean temperature of wettest quarter	°C	Sw	Swamp/Mangrove	sq. km.
bio9	Mean temperature of driest quarter	°C	Gr	Grass/grazing land	sq. km.
bio10	Mean temperature of warmest quarter	°C	Sn	Snow	sq. km.
bio11	Mean temperature of coldest quarter	°C	InW	Inland wetland	sq. km.
bio12	Annual precipitation	mm	CoW	Coastal wetland	sq. km.
bio13	Precipitation of wettest month	mm	Ri	River/stream/canals	sq. km.
bio14	Precipitation of driest month	mm	WaB	Waterbodies	sq. km.
bio15	Precipitation seasonality (coefficient of variation)	mm	Land1**	Land cover PCA axis 1	
			Land2**	Land cover PCA axis 2	
bio16	Precipitation of wettest quarter	mm	Land3**	Land cover PCA axis 3	
bio17	Precipitation of driest quarter	mm	Land4**	Land cover PCA axis 4	
bio18	Precipitation of warmest quarter	mm	**Socioeconomics**		
bio19	Precipitation of coldest quarter	mm	HumPopDens	Population density	sq. km.
Climate1*	Climate PCA axis 1		UrPop	Urban population	%
Climate2*	Climate PCA axis 2		RuPop	Rural population	%
Climate3*	Climate PCA axis 3		LitRate	Literacy rate	%
Climate4*	Climate PCA axis 4		GDPpercap	Gross domestic product per Capita	USD
			Livestock	Total Livestock	million

To obtain information on butterfly life histories, we tabularised for all species the following information: (i) wing span (numeric, mm) as a simple measure of body size, potentially related to adult mobility (Sekar 2012), but also to development length and host plants' defences (Bartonova et al. 2014); (ii) larval host plant growth form (categorical, distinguishing forbs, grasses, tall grasses, shrubs, climbers/vines, trees and ants‐dependent/carnivorous), which is straightforwardly linked to habitat use (Jain et al. 2017) and to host plants' defences and butterfly voltinism (Altermatt 2010a); (iii) larval plants' forms scope (numeric), obtained as a simple sum of larval host plant growth forms, assuming that broader dietary range links to more generalised resource use (Shreeve et al. 2001; Vanreusel and Van Dyck 2007); (iv) distribution range in India, defined as the number of states with positive occurrence (numeric, 1–36); (v) global distribution range (ordinal at 1–5 scale, where 1—particular region, ideally smaller than the area of India, 2—size of India, 3—greater than India but smaller than Indo‐Malayan realm, 4—greater than Indo‐Malayan realm or size of a continent, 5—Cosmopolitan). For missing values (*n* = 464, i.e., 3.04% of the 15,246 cells of the traits' matrix, all related to host plant forms), we used arithmetic means of relevant columns of the table. Note that (iv + v) does not represent life history traits in a strict sense but convey information on niche breadth and potential distribution areas (Alzate and Onstein 2022; Dewan et al. 2022; Hausharter et al. 2023).

Because species are not independent units but share phyletic histories, relations between life history traits and their distributions should be constrained by phylogeny in analyses (Harvey and Pagel 1991; Seifert and Fiedler 2024). We used the phylogeny tree and its list of all butterflies from Kawahara et al. (2023). From this, we manually selected all the genera distributed in India and removed the remaining genera using the ‘keep.tip’ function from the R package *ape* (Paradis and Schliep 2019). The genera not included in the phylogeny tree were added to the closest proximate node manually by following available individual phylogeny using the function ‘bind.tip’. Species not represented in the tree (*n* = 1094) were added to the roots of their respective genera using the ‘add.species.to.genus’ (R package *phytools*) (Revell 2024), while species not occurring in India were removed from the Indian genera root by utilising the ‘drop.tip’ function. See Table S1 for the final species list.

### 2.2 Data Analyses

Prior to analyses, we transformed all predictors describing individual states to zero means and unit variances to facilitate mutual comparability of their effects. Species data per individual states entered the analyses in 1/0 form. The analyses were carried out in CANOCO v.5.1 (ter Braak and Smilauer 2018). Significances of constrained ordinations were assessed using 999 Monte Carlo permutations.

We first subjected the 19 *climate* and 24 *land cover* predictors to two separate principal component analyses (PCA) centred on the variables, with the 36 states treated as samples. The obtained principal components describing the variation of *climate* and *land cover* across Indian federal states were used in subsequent analyses (Figures S2 and S3).

Second, to visualise similarities of butterfly faunas of individual states, we run PCA (centred by species), again with the 36 states as samples and respective species' lists as response variables. Third, we computed four constrained linear redundancy analyses (RDA) based on *geography*, *climate*, *land cover* and *socioeconomic* predictors (including second‐order interactions and quadratic functions in case of *geography* coordinates), selecting appropriate combinations of predictors via the CANOCO forward selection procedure. We then used the selected terms of the constrained ordinations as covariables in partial unconstrained (pPCA) and partial constrained (pRDA) analyses used to quantify mutual effects of the four groups of predictors on the pattern in species distribution.

Finally, we interpreted the PCAs and RDAs derived patterns by life history traits. We used the fourth‐corner approach (Legendre et al. 1997; Dray and Legendre 2008), which relates results of ordinations of two tables (here, states × species) to a third table (the traits). Technically, this was done via two‐step ordinations, explaining axes from the previous PCA and RDA analyses by the traits using RDAs with 999 Monte‐Carlo permutations.

To investigate the effects of phylogeny on the axes–trait patterns, we used the phylogeny tree to generate a square dissimilarities matrix (in R, using the function ‘cophenetic.phylo’) and subjected it to the principal coordinate analysis (PCO) in CANOCO. These generated 1386 PCO vectors, by which we constrained the RDA results, manually selecting only PCO vectors explaining > 2.0% of variation in the species occurrences data. We then interpreted the thus constrained RDA axes by life history traits, as in analyses not constrained by phylogeny.

## 3 Results

**FIGURE 1:**
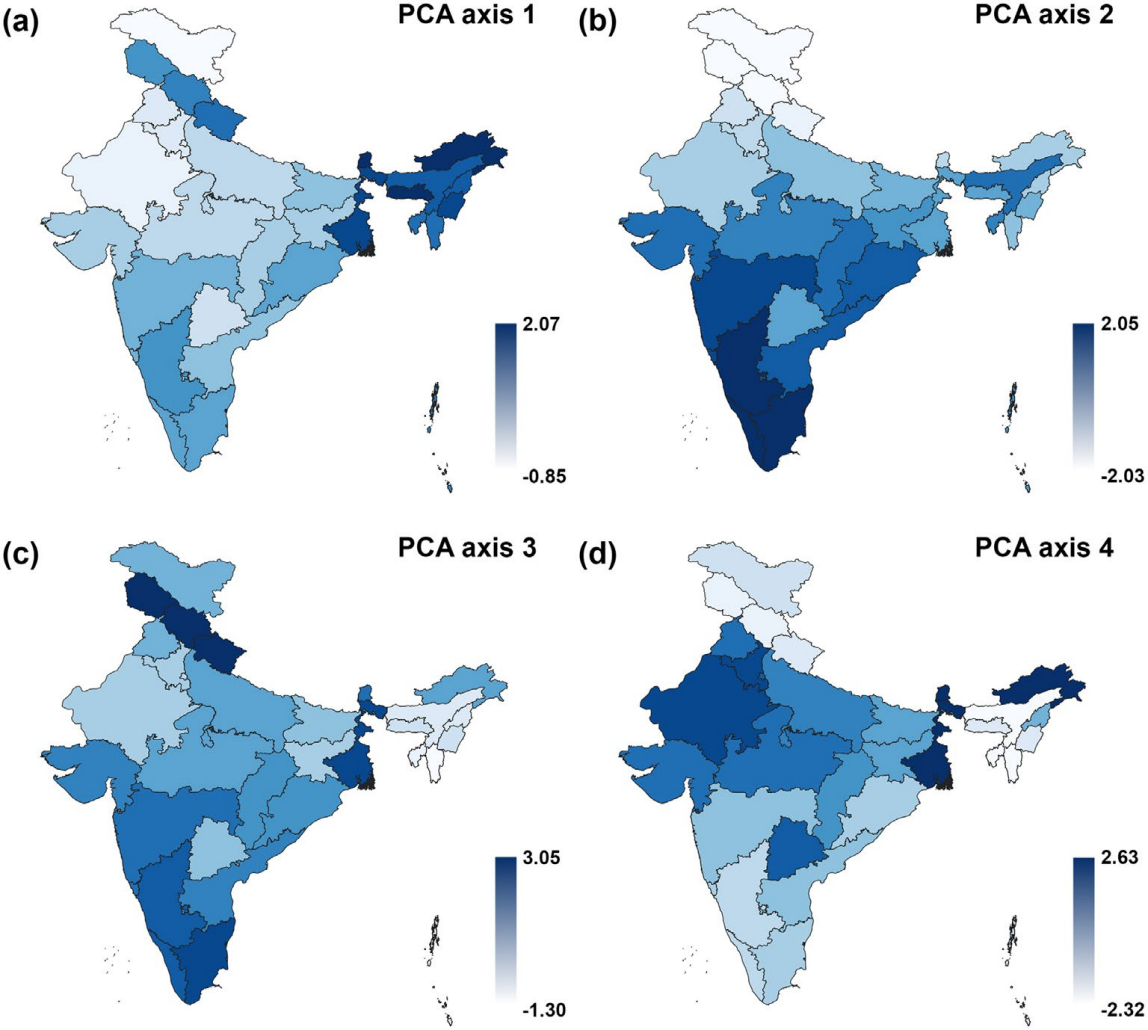
Visualisation of results of PCA analysis, relating occurrences of 1386 butterfly species recorded from the 36 federal states and union territories of the Republic of India. Intensity of colours is proportional to values of loadings at the 1st–4th PCA axes.

The unconstrained PCA relating butterflies to federal states revealed prominent geography patterns (Figure 1). The first principal component (eig 0.389) separated humid monsoon northeastern states from the rest; the second component (eig 0.121) separated the southern peninsular states from those in the Himalayan North of the peninsula; the third (eig 0.088) distinguished Himalayan and southern mountainous (Western Ghats) states from both the northeast and interior of the peninsula, while the fourth (eig 0.040) accounted for only a minor part of the variation.

The partial pPCAs indicated that *geography* had overwhelming effects on the distribution of butterflies, explaining 62% of variation in the data. This was followed by *climate* (36%), *land covers* (29%) and *socioeconomics* (18%) (Table 2). Visualising the results showed that with *geography* as covariables, the relationships among states completely dissipated, whereas in the models controlled for *climate*, *land covers* and *socioeconomics*, the basic patterns (northeast vs. the rest, Himalayas vs. the rest) remained preserved (Figure S4).

**TABLE 2:** Results of unconstrained (PCA) and constrained (RDA) ordinations explaining the butterfly species composition at the level of 36 federal states and union territories of the Republic of India. The combinations of *geography*, *climate*, *land covers* and *socioeconomics* predictors were obtained by forward selection procedures, the same combinations were used in partial ordinations as covariables (notified as ‘|’ in the table). The models marked by ‘^+^’ were used in subsequent third‐order analyses with life history traits (Table 3).

Model	Model structure	Var_adj_	Eig1	Eig2	Eig3	Eig4	*F* ^*p*^_axis1_	*F* ^*p*^_all axes_
Unconstrained (PCA)^+^	~		0.389	0.121	0.088	0.040		
~|Geography	~|*x* + *y* + x*y + x*z + y*z + *y* ^2^ + *z* ^2^ + AltDiff	62.1	0.074	0.048	0.031	0.024		
~|Climate	~|Climate1 + Climate2 + Climate3	36.3	0.158	0.073	0.064	0.036		
~|Land covers	~|Land1 + Land3	29.6	0.187	0.106	0.064	0.036		
~|Socioeconomics	~|Rural population + Livestock	18.4	0.258	0.108	0.079	0.038		
Geography RDA^+^	*x* + *y* + x*y + x*z + y*z + *y* ^2^ + *z* ^2^ + AltDiff	50.9	0.338	0.095	0.079	0.032	1.7***	5.5***
~|Climate	*y* + x*z + y*z + *z* ^2^ + AltDiff|Climate	26.1	0.107	0.054	0.038	0.026	1.1***	3.3***
~|Land covers	*x* + *y* + x*y + x*z + y*z + *y* ^2^ + *z* ^2^ + AltDiff|Land covers	37.5	0.143	0.076	0.057	0.027	0.8***	3.5***
~|Socioeconomics	*x* + *y* + x*y + x*z + y*z + *y* ^2^ + *z* ^2^ + AltDiff|Socioeconomics	42.0	0.210	0.078	0.071	0.029	1.1***	4.0***
Climate RDA^+^	Climate1 + Climate2 + Climate3	30.3	0.281	0.063	0.018	—	4.2***	6.1***
~|Geography	–|Geography	—	—	—	—	—	—	—
~|Land covers	Climate1 + Climate2 + Climate3|Land1 + Land3	18.3	0.111	0.047	0.023	—	1.9***	3.5***
~|Socioeconomics	Climate1 + Climate2|Socioeconomics	20.2	0.157	0.047	—	—	3.7***	5.2***
Land covers RDA^+^	Land1 + Land3	25.4	0.230	0.066	—	—	4.9***	6.9***
~|Geography	Land3|Geography	03.9	0.028	—	—	—	2.1**	—
~|Climate	Land1 + Land3|Climate	12.5	0.071	0.043	—	—	1.9**	3.3***
~|Socioeconomics	Land1 + Land3 | Socioeconomics	15.1	0.112	0.054	—	—	2.5***	3.9***
Socioeconomics RDA^+^	Rural population + Livestock	13.5	0.160	0.025	—	—	3.1**	3.7**
~|Geography	–|Geography	—	—	—	—	—	—	—
~|Climate	–|Climate	—	—	—	—	—	—	—
~|Land covers	–|Land covers	—	—	—	—	—	—	—

*Note:* Monte‐Carlo permutation tests significance values: **p < 0.01, ***p < 0.001.

**FIGURE 2:**
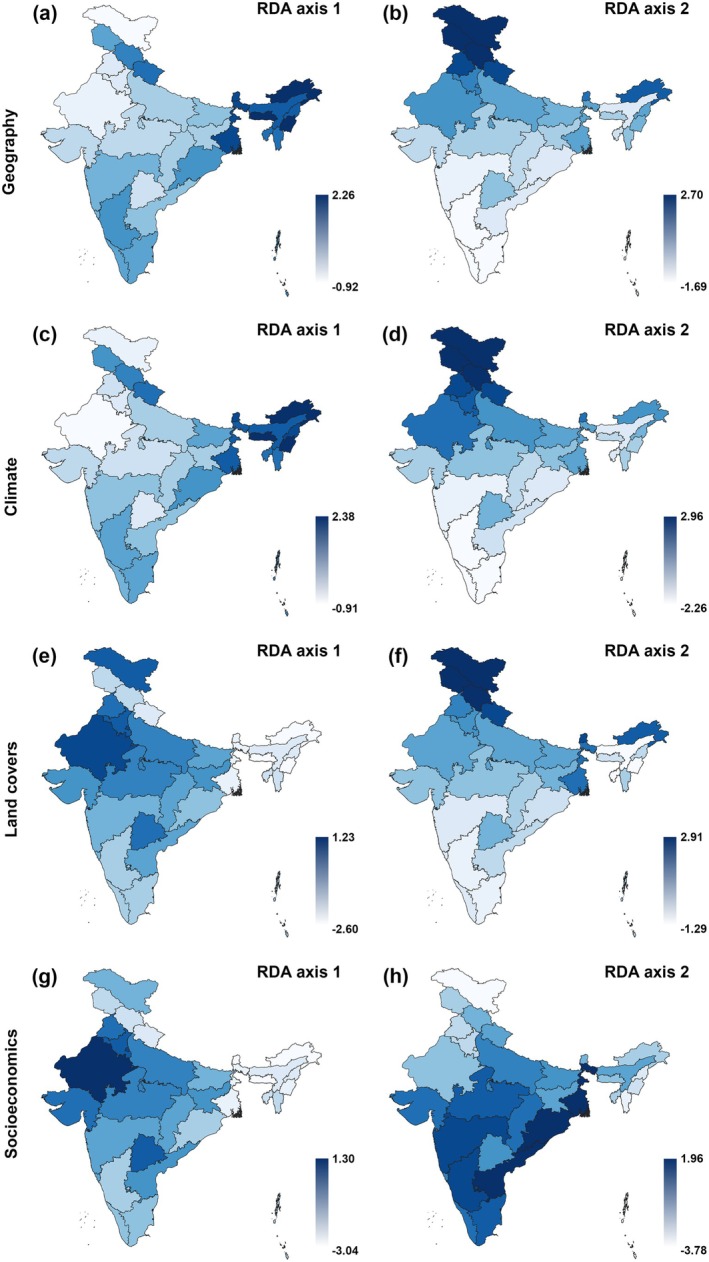
Visualisation of results of the (constrained) RDA analyses, relating occurrences of 1386 butterfly species recorded from the 36 federal states and union territories of the Republic of India to *geography*, *climate*, *land covers* and *socioeconomics*. See Table 2 for terms of the ordination models and relevant statistics. Intensity of colours is proportional to values of loadings at the 1st and 2nd RDA axes of each model.

The constrained RDA ordinations (Table 2, Figure 2) corroborated that the strongest predictor of per‐state butterfly species composition was *geography*, followed by *climate*, *land covers* and *socioeconomics*. Consequently, controlling *climate*, *land covers* and *socioeconomics* models by *geography* resulted in non‐significant models. *Climate*, however, retained some independent effects if controlled by *land covers* and *socioeconomics*; *land covers* retained a weak independent effect if controlled by *climate* and *socioeconomics*, and *socioeconomics* did not sustain controls by other sets of predictors.

**FIGURE 3:**
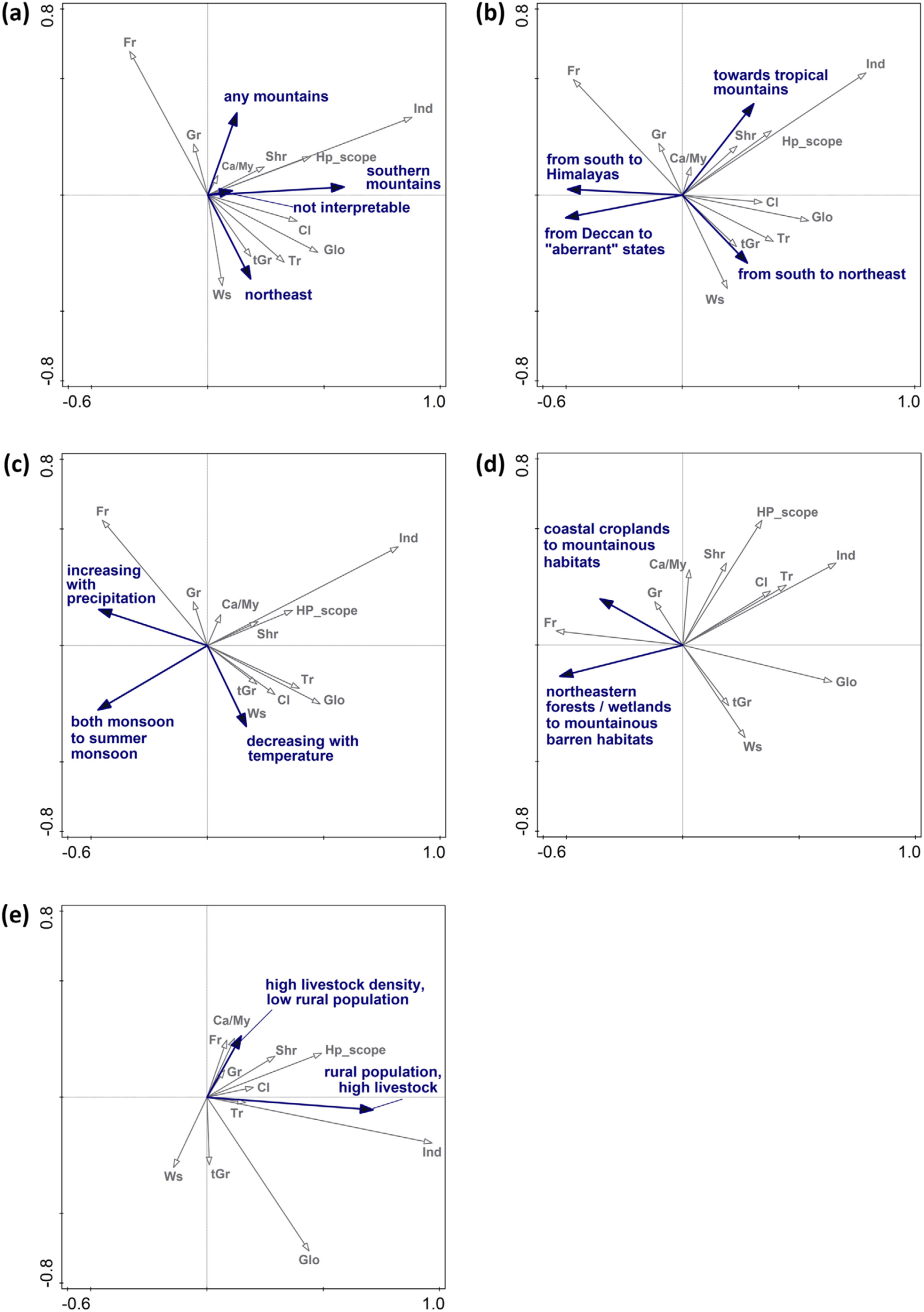
Ordination diagrams obtained by the third‐corner analyses (axes 1st and 2nd), using RDA to relate the ordination axes (blue darts) from unconstrained PCA (a) and constrained RDA analyses (constrained by b: *geography*, c: *climate*, d: *land covers*, e: *socioeconomics*), to life history traits (grey darts) of the butterfly species recorded in the 36 federal states and union territories of the Republic of India. See Tables 2 and 3 for the relevant statistics. Traits abbreviations: Ca/My, carnivorous/ants‐dependent or Myrmecophily; Cl, climbers/vines; Fr, (larval development on) forbs; Glo, global range; Gr, grasses; Hp_scope, larval host plants scope; Ind, Indian distribution range; Shr, shrubs; tGr, tall grasses; Tr, trees; Ws, wing span.

Interpreting the ordination results by life history traits (Table 3) revealed that in unconstrained analysis, large‐bodied butterflies developing on trees, climbers or tall grasses inclined towards northeastern states (Figure 3a). Such butterflies also tend to have wide distributions across the Old World tropics. Species developing on shrubs or having wide host plant scope inclined towards southern mountainous states. Finally, small‐bodied species developing on grasses and forbs inclined towards mountainous states. The patterns obtained by interpreting the *geography* model were very similar (Figure 3b). The patterns for *climate* again showed the association of large‐bodied butterflies with development on climbers, trees, tall grasses, and shrubs, and the association of all these traits related to monsoon climates with high precipitation and high temperatures around the Indian Peninsula. Such butterflies also inhabit large distribution ranges globally and occur in many Indian federal states. Small‐bodied butterflies developing on forbs or short grasses displayed the opposite patterns, that is, an association with high temperatures and precipitation (Figure 3c). For *land covers*, the large‐bodied butterflies associated with tall grasses inclined towards states containing coastal croplands. They also inhabit large ranges. In contrast, high representation of forests was associated with development on shrubs, trees, climbers and occurrence in many Indian states. Small butterflies developing on forbs were situated oppositely to any trees in mountainous states (Figure 3d). Interpreting the *socioeconomics* ordination was most puzzling, as it produced a very strong first ordination axis (cf. Table 3), parallel with high rural population and high livestock. These conditions were associated with wide host plant scopes, feeding on shrubs, distribution in many Indian states, relatively small wing spans and rather wide global ranges. The much weaker second ordination axis ran in parallel with low rural population and high livestock, conditions typical for sparsely populated arid areas. Here, a high representation of small‐bodied butterflies develops on forbs or grasses. The large‐bodied butterflies, typically feeding on tall grasses, were most represented in states with low rural population and low livestock, that is, in the densely forested states (Figure 3e).

**TABLE 3:** Statistical properties of RDA models, using third‐corner approach to relate the ordination axes from models relating the distribution of 1386 butterfly species in 36 Indian federal states and union territories to predictors describing the states (summarised in Table 2), to life history traits of the butterflies. The models are visualised at Figures 3 and 4.

Model	Var_adj_	Eig1	Eig2	Eig3	Eig4	*F* ^*p*^_axis1_	*F* ^*p*^_all axes_
*Ignoring phylogeny*							
Unconstrained (PCA)	18.0	0.101	0.062	0.013	0.008	15.5***	31.3***
Geography RDA	22.4	0.156	0.061	0.010	0.002	25.3***	40.9***
Climate RDA	23.7	0.156	0.074	0.013	—	25.4***	44.1***
Land covers RDA	22.6	0.203	0.029	—	—	34.9***	41.4***
Socioeconomics	29.6	0.265	0.036	—	—	49.6***	59.1***
*Considering phylogeny*							
Unconstrained (PCA)	11.1	0.056	0.012	0.005	0.003	12.0***	16.8***
Geography RDA	13.0	0.065	0.007	0.004	0.001	16.2***	19.8***
Climate RDA	10.5	0.047	0.011	0.004	0.002	11.3***	15.8***
Land covers RDA	11.2	0.048	0.009	0.002	0.001	12.8***	16.3***
Socioeconomics RDA	17.7	0.088	0.014	0.005	0.001	22.2***	28.3***

*Note:* Monte‐Carlo permutation tests significance values: **p < 0.01, ***p < 0.001.

**FIGURE 4:**
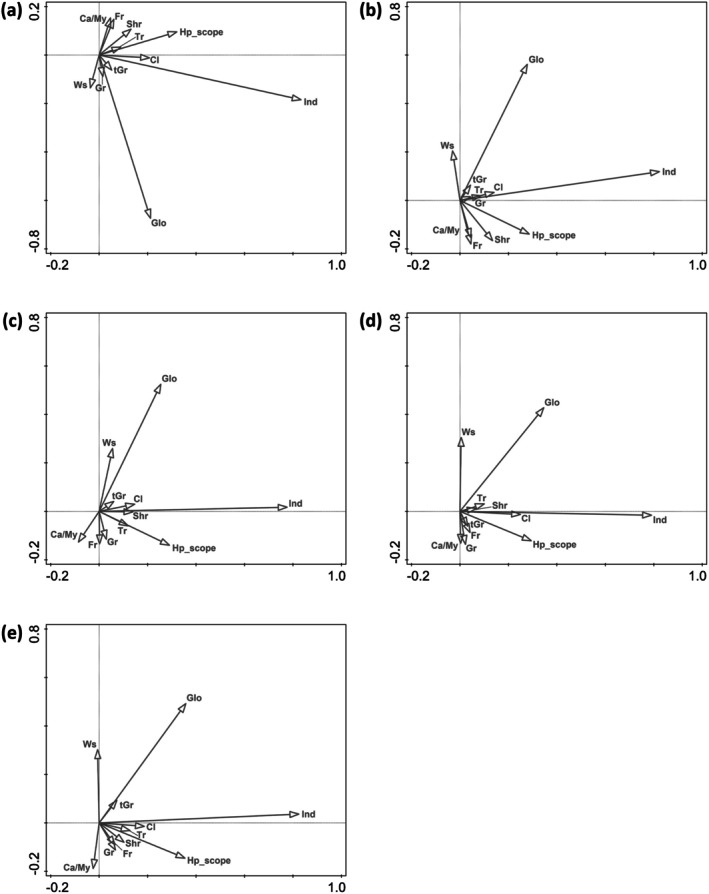
Ordination diagrams obtained by the butterfly phylogeny constrained third‐corner analyses (axes 1st and 2nd), using RDA to relate the ordination axes from unconstrained PCA (a) and constrained RDAs (constrained by b: *geography*, c: *climate*, d: *land covers*, e: *socioeconomics*), to life history traits of the butterfly species recorded in the 36 federal states and union territories of the Republic of India. Only the relationships among traits (grey darts) are shown. See Tables 2 and 3 for the relevant statistics. Traits abbreviation: Ca/My, carnivorous/ants‐dependent or Myrmecophily; Cl, climbers/vines; Fr, (larval development on) forbs; Glo, global range; Gr, grasses; Hp_scope, larval host plants scope; Ind, Indian distribution range; Shr, shrubs; tGr, tall grasses; Tr, trees; Ws, wing span.

The models considering butterfly phylogeny explained much lower amounts of variation than the models ignoring it (Table 3). The difference in explained variation between the models ignoring and considering phylogeny was highest for *socioeconomics*, implying that patterns of butterfly distribution explicable by socioeconomic conditions were the least phylogeny dependent. Interpreting the models by traits dissolved the associations between body size, feeding on trees and global distribution, while retaining the associations between feeding on shrubs and climbers with a high number of host plant forms (Figure 4a). The link among global distribution, the number of inhabited Indian states, and the number of host plant forms was retained in the analysis constrained by *geography* (Figure 4b), while body size lost the association with feeding on trees, implying a strong phylogenetic dependency of large bodies with development on tree foliage. The patterns were almost identical in analyses with *climate*, *land cover* and *socioeconomics* (Figure 4c–e).

## 4 Discussion

Using multivariate analysis to explore the distributional patterns of butterfly species at the level of 36 federal states and territories forming the Republic of India, we found a significant effect of geography, which explained over 60% of the variation in the analysis constrained by geographic predictors. Climate, land covers and socioeconomic characteristics of the federal states had less significant explanatory effects and were closely tied to geography. The patterns of species distribution were attributable to their life histories. Large‐bodied species inclined towards northeastern states with a warm and humid monsoon climate, dense forests and low representation of the rural population around the Indian Peninsula. The growth forms of larval host plants reflected a broad distribution of plant growth forms in the main biomes of India. Larval development on trees, climbers and tall grasses corresponded with occurrence in states situated in the humid forest biome, and development on forbs and grasses with states containing arid or alpine biomes. The scope of larval host plant forms used by individual species increased with feeding on shrubs and climbers, and butterflies with broad host plant form scopes inhabited high numbers of federal states and displayed wide global distributions. Despite the still limited information on the life histories of Indian butterflies, species' traits were linked to their ecology and distribution.

The significant geography effect and existence of geographically linked faunal structures were previously observed by Holloway (1974), who used the best numeric method available at his time, a cluster analysis on a selection of butterfly genera. Das et al. (2023) developed this approach in their analysis of butterfly species counts across Indian federal states. The results presented here corroborate the earlier findings using species identities.

The effect of geographic predictors is attributable to the peculiar geography of the Indian peninsula, which affected the phyletic history of Indian taxa. In the Cretaceous period, when the basal radiation of Papilionoidea occurred in Gondwanan South America, Australia and the eastern parts of the Northern continent (Kawahara et al. 2023), the Indian peninsula was a large island, gradually drifting northwards after the breakup of Gondwana. Not too high diversity of indigenous lineages is expectable on islands (MacArthur and Wilson 1967) and the diversity was possibly affected by late‐Cretaceous catastrophic volcanism of the Deccan traps (cf. Wellman and McElhinny 1970; Schoene et al. 2015). Later, the colonisation of the current peninsula by butterfly lineages occurred mainly from the archipelagos now forming the eastern part of the Indo‐Malayan zoogeographic realm, which communicated with both Australian and East‐Palearctic regions (cf. Kawahara et al. 2023). The peninsular India, however, had been and remains relatively isolated from the eastern parts of the Indo‐Malayan region. Only a narrow conduit formed by the Garo‐Rajmahal gap in present‐day Bangladesh connects it with more easterly tropical areas (Tiwari and Jassal 2001; Das et al. 2023), while the Himalayas block the communication with the Palearctic (Mani 1986). The colonisation from Africa, via transoceanic transfers or overland, had always been restricted (Kodandaramaiah and Wahlberg 2007; Irungbam et al. 2023). Consequently, the fauna of the federal states east of the Garo‐Rajmahal gap is richer than the fauna of peninsular India (Holloway 1974; Kunte et al. 2012). A notable exception is the Western Ghats states in SW India, which form a secondary speciation and endemism centre (Gaonkar 1996; Kunte 2016), but also display increased representation of afro‐tropical species (Das et al. 2023).

Climatic patterns are fundamentally governed by geography, which is evident from the effects of coasts and major mountain ranges on monsoons (Lu et al. 2020; Chauhan et al. 2023), or from differences in vegetation between windward and leeward mountain slopes (Pepin et al. 2017). Climate, however, directly affects the eco‐physiological tolerance of insects (Sinclair et al. 2003; Vrba et al. 2017). Das et al. (2023) showed that per‐state richness of Indian butterflies reflects the available energy, in agreement with various taxa from across the world (e.g., Wright 1983; Hawkins and Porter 2003; Carrara and Vázquez 2010; Duncan et al. 2015). The two main climatic connections influencing the species composition of Indian states' faunas are that connecting the mountainous and humid northeast with northwestern Himalayan and western Ghats states (shared species, e.g., *Celastrina lavendularis*, *Neptis nata*), and that connecting the trans‐Himalayan Ladakh with sparsely forested arid states of NW peninsula and central Deccan (e.g., *Gegenes nostradamus*, *Belenois aurota*).

In agreement with our original prediction, land covers and socioeconomic predictors executed weaker influences on the composition of Indian states' faunas, and even their influences reflected underlying effects of geography (e.g., low forest cover in the high mountainous Ladakh) or climate (e.g., the high livestock numbers in the arid Rajasthan, the warm and humid densely forested northeastern states). Consequently, the land covers produced ordinations very similar to climate and geography, whereas the analysis based on socioeconomic predictors produced completely different patterns, decoupled from the geography‐based faunal structures. Recall that the weak effect of socioeconomic predictors relates to areas of entire states; it might become much stronger at finer scales, as suggested by studies of urbanisation effects on insects (Diamond et al. 2023).

Given incomplete knowledge of life histories of many Indian species (cf. Shirey et al. 2022), we tabularised only a few readily available traits. Still, the distribution of the traits in federal states' faunas reflected highly significantly the predictors describing the states. The most easily available trait, the wing span (a body size proxy), increased towards northeastern states, and the *climate*, *land covers* and *socioeconomics* models all agreed that forests of Indian northeast host the largest butterflies. Indeed, some of the largest species of Indian fauna are either restricted to northeastern states (e.g., *Atrophaneura aidoneus*, *Papilio paradoxa*, *Stichophtalma sparta*), or occur there and in other humid forested regions (e.g., *Troides aeacus*, *Byasa dasarada*, *Papilio arcturus*). By supplement, small‐bodied butterflies were associated with mountainous and/or northwestern states, including the trans‐Himalayan Ladakh. Some of the smallest Indian butterflies are restricted to the latter (e.g., *Turanana chitrali*, *Agriades pheretiades*, *Pyrgus alpinus*), which does not exclude the presence of some small species in tropical peninsular India (e.g., *Freyeria putli*, *Zizula hylax*, *Spialia galba*).

The debate on the geographic distribution of butterfly (or Lepidoptera) body sizes has long been obscured by inconsistent results from different continents and clades, suggesting effects of phyletic history (Barlow 1994; Hawkins and Lawton 1995). A trade‐off exists between body size and development speed, and hence the number of generations (Seifert et al. 2023). In high latitudes, lepidopteran body size—voltinism relationships are restricted by season length, available temperatures and the ecosystems' net primary productivity (Huston and Wolverton 2011; Zeuss et al. 2017). Similar constraints likely restrict butterfly body size in mountainous or arid regions, including those of India. In the highly productive rainforest states (cf. Das et al. 2023), the presence of large‐sized butterflies is fully expected.

In ordinations of species traits, large wingspan corresponded with development on tall grasses (i.e., bamboos of the genera *Bambusa*, *Dendrocalamus*, *Saccharum*), climbers (e.g., *Aristolochia*, *Cynanchum*, *Vincetoxicum*) and trees, whereas small wingspan corresponded with development on small grasses and forbs. This appears linked to prevailing land covers in the respective states (humid forests in the northeastern, Himalayan slopes and Western Ghats states; grasslands in arid northwest and trans‐Himalayan Ladakh). It does not explain, however, why the rainforest butterflies tend to grow large, rather than staying small and forming multiple generations. Young plant tissues are more palatable for young herbivorous insect larvae than old tissues (Cizek 2005). Then, development on woody plants requires that the larvae start feeding on fresh foliage, which constraints the numbers of insect generations while favouring larger final size (Cizek et al. 2006; Altermatt 2010b; Seifert et al. 2023). Hatada and Matsumoto (2007) observed that larval development of the papilionid *Luehdorfia japonica* was longer on old than young leaves. Because availability of young plant tissues is seasonally less predictable in humid tropics than in seasonal regions, the herbivores, including butterflies, may be selected for larger body size, and hence the ability to develop on older tissues. The relationships between development length, body size and voltinism are further linked to the plants' antiherbivore defences (Smilanich et al. 2016), which remain little explored in the tropics (but see Endara et al. 2017; Segar et al. 2024).

Host plant scope, expressed here as the number of host plant growth forms utilised per species, was related to larval development on shrubs, climbers and less so trees and tall grasses, but not small grasses and forbs. Notably, some genera of Indian flora contain trees, shrubs and climbers (e.g., *Morinda*, *Ficus*), but not forbs and small grasses. This implies that the basic distinction between species developing on small/unapparent and qualitatively protected plants, typically forbs, versus large/apparent and quantitatively protected plants, that is, trees, shrubs and tall grasses (Feeny 1976), exists in Indian butterfly fauna. The host plant scope as used by us might also be related to taxonomic trophic range (i.e., the number of host species), and hence to niche breadth and commonness vs. rarity. Such relationships are unlikely straightforward, as common but strictly monophagous butterflies exist, for example, in the Palearctic (cf. Bryant et al. 1997). Still, broad host plant scope is positively linked to latitudinal range size in Europe (Seifert and Fiedler 2024). The wide host scope was also collinear with the number of Indian states inhabited, suggesting that such butterflies inhabit many federal states, including species‐poor central peninsular areas and small coastal territories. It was also collinear with wide global distribution. Indian butterflies displaying these trait combinations include such widespread pantropical species as *Hypolimnas missipus*, *Lampides boeticus* or *Eurema blanda*.

In analyses controlled for phylogeny, the relationship between large body size and feeding on trees or tall grasses dissipated. This reflects the existence of species‐rich clades, whose members are similar in size and conserved in associations with certain host plant forms. Examples at the large‐bodied side include *Graphium* or *Euthalia* developing on trees (22 and 20 species in India, respectively); *Byasa* and *Euploea* developing on vines (7 and 12 species) or *Lethe* developing on tall grasses (42 species). At the small‐bodied side, *Heliophorus* (12 species) and *Jamides* (8 species) all develop on small forbs.

In contrast, the positive relationships between the number of host plant forms, numbers of Indian states and global distribution were independent of phylogeny, suggesting that broad larval trophic ranges covary with wide distributions independently of the phylogeny of such successful taxa.

To conclude, we demonstrated that analysing factors affecting regional species compositions is possible for tropical faunas even at a relatively crude level of political units, such as Indian federal states and territories. Geography crucially affects the composition of regional faunas, and interpretation of the faunal compositions by life history traits revealed intriguing patterns. The number of traits analysed, however, was severely restricted by still incomplete knowledge of life histories of many of the Indian species. Therefore, much work aiming to decipher detailed ecological requirements of individual species is needed.

## Author Contributions

**Gaurab Nandi Das:** conceptualization (equal), data curation (lead), formal analysis (equal), methodology (equal), visualization (equal), writing – original draft (equal). **Zdenek Faltynek Fric:** conceptualization (equal), formal analysis (equal), methodology (equal), visualization (equal), writing – review and editing (equal). **Martin Konvicka:** conceptualization (equal), formal analysis (equal), methodology (equal), supervision (lead), visualization (equal), writing – original draft (equal), writing – review and editing (equal).

## Conflicts of Interest

The authors declare no conflicts of interest.

## Supporting information


**Data S1:** ece372217‐sup‐0001‐DataS1.docx.

## Data Availability

This study used species distribution data available in ‘Dryad’ at https://doi.org/10.5061/dryad.mpg4f4r9r.
