# Supplementary material for: Geography of Life Histories in a Tropical Fauna: The Case of Indian Butterflies
Source: Ecol Evol. 2025 Sep 22;15(9):e72217. doi: 10.1002/ece3.72217 (PMC12453613; doi:10.1002/ece3.72217)
Supplement: Supplementary file 1 — Data S1: ece372217‐sup‐0001‐DataS1.docx. [file ECE3-15-e72217-s001.docx]

Supplementary information for

**Geography of life histories in a tropical fauna: The case of Indian butterflies**

Gaurab Nandi Das, Zdenek Faltynek Fric, Martin Konvicka

This file includes

FIGURE S1. Key to Indian states with their butterfly species richness.

Unconstrained ordination of bioclimatic and land covers variables.

FIGURE S2. Unconstrained ordination of bioclimatic variables.

FIGURE S3. Unconstrained ordination of land covers variables.

FIGURE S4. Visualisation of results of partial pPCA analyses.

TABLE S1. Details on preparation of phylogeny of Indian butterflies.

**
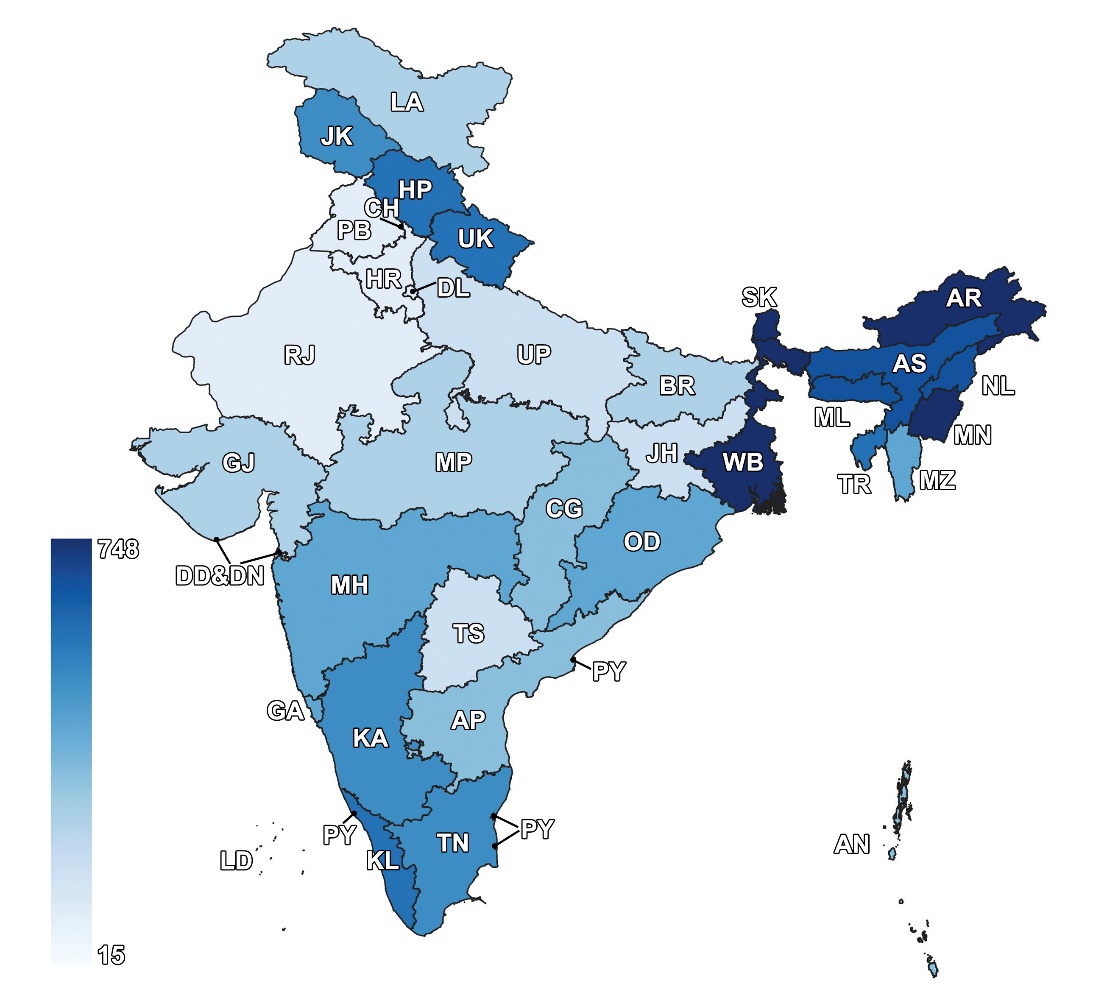
**

FIGURE S1. Key to Indian states with their butterfly species richness.

State abbreviations: AN – Andaman and Nicobar, AP – Andhra Pradesh, AR – Arunachal Pradesh, AS – Assam, BR – Bihar, CG – Chhattisgarh, CH – Chandigarh, DD & DN – Daman and Diu and Dadra and Nagar Haveli, DL – Delhi, GA – Goa, GJ – Gujarat, HP – Himachal Pradesh, HR – Haryana, JH – Jharkhand, JK – Jammu and Kashmir, KA – Karnataka, KL – Kerala, LA – Ladakh, LD – Lakshadweep MH – Maharashtra, ML – Meghalaya, MN – Manipur, MP – Madhya Pradesh, MZ – Mizoram, NL – Nagaland, OD – Odisha, PB – Punjab, PY – Puducherry, RJ – Rajasthan, SK – Sikkim, TN – Tamil Nadu, TR – Tripura, TS – Telangana, UK – Uttarakhand, UP – Uttar Pradesh, WB – West Bengal.

The colour scale is proportional to butterfly species richness.

**Unconstrained ordination of bioclimatic and land covers variables**

We first subjected the 19 climate and 24 land covers predictors to two separate principal component analyses (PCA) centered on the variables, with the 36 states treated as samples. The obtained principal components describing the variation of climate and land covers in Indian federal states, were used in subsequent analyses.

For climate, the principal components were Climate1 (eigenvalue [eig] 0.433, decreasing with temperature); Climate2 (eig 0.313, increasing with precipitation); Climate3 (eig 0.122, from states with both summer and autumn monsoon to those with summer monsoon only); and Climate4 (eig 0.061, from regions with high climatic seasonality to those with high diurnal differences).

For land covers, these were Land1 (eig 0.216, from coastal swampy states with high representation of cropland to mountainous states with high representation of evergreen forests, alpine habitats and much shifting agriculture); Land2 (eig 0.199, from mountainous or arid states with barren land, rugged terrain and scrub towards highly urbanised states with high representation of deciduous forests and plantations); Land3 (eig 0.124, from northeastern states with evergreen forests, inland wetlands, large rivers and rural landscapes to Himalayan states with barren, sandy or alpine habitats); and Land4 (eig 0.099, from highly urbanised states with a large representation of cropland, to coastal/island areas with coastal habitats, swamps, and plantations).


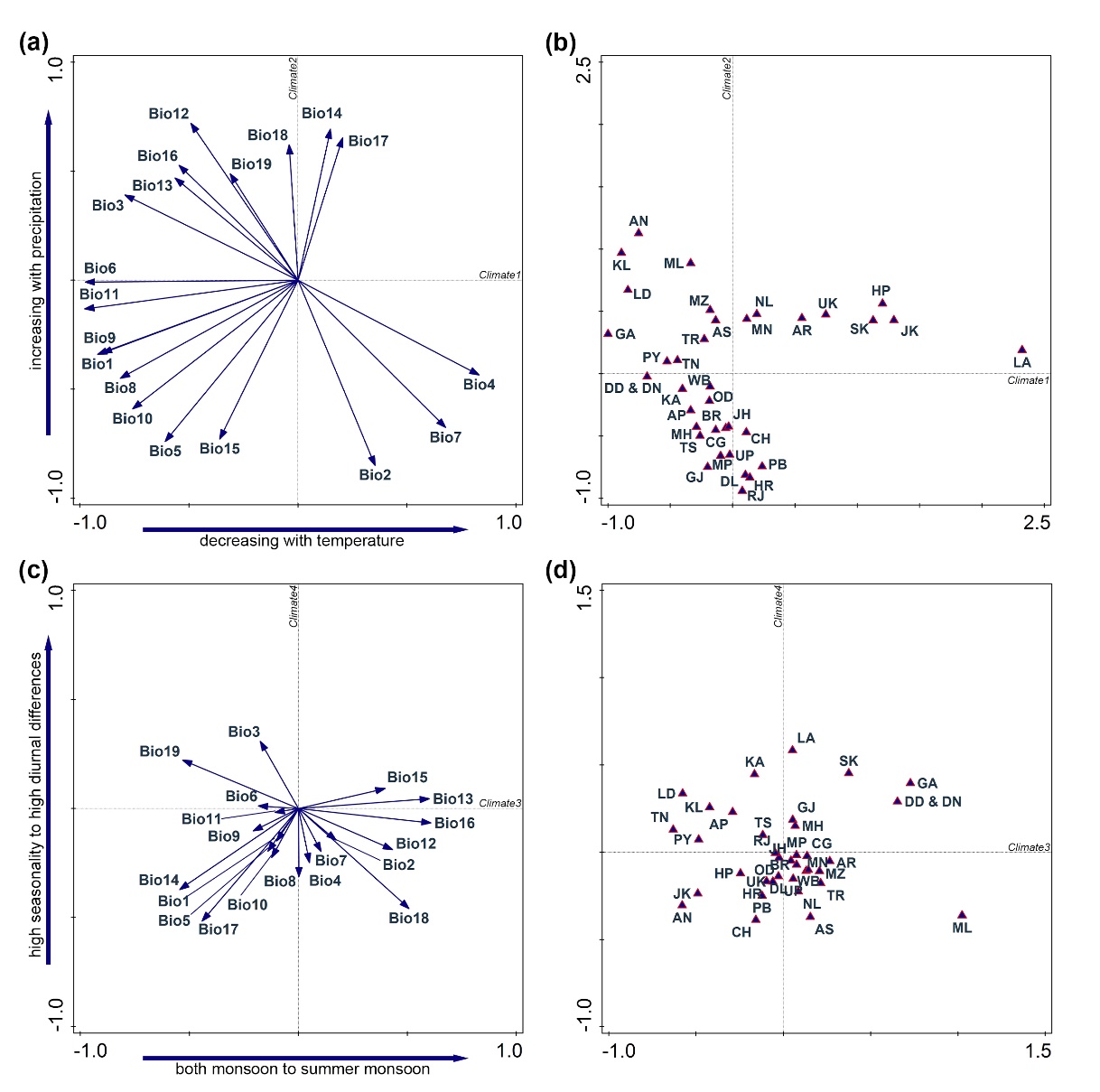


FIGURE S2. Unconstrained ordination of bioclimatic variables

PCA biplots showing the mutual positions of 19 bioclimatic variables at the first and the second ordination axes (a), positions of Indian federal states along first and second axes (b), positions of the variables at the third and fourth ordination axes (c), and positions of Indian federal states along the third and fourth axes (d).

Key, bioclim variables (from WorldClim 2024): Bio1 – Annual Mean Temperature, Bio2 – Mean Diurnal Range (Mean of monthly max temp – min temp), Bio3 – Isothermality (Bio2/Bio7), Bio4 – Temperature Seasonality, Bio5 – Max Temperature of Warmest Month, Bio6 – Min Temperature of Coldest Month, Bio7 – Temperature Annual Range (BIO5–Bio6), Bio8 – Mean Temperature of Wettest Quarter, Bio9 – Mean Temperature of Driest Quarter, Bio10 – Mean Temperature of Warmest Quarter, Bio11 – Mean Temperature of Coldest Quarter, Bio12 – Annual Precipitation, Bio13 – Precipitation of Wettest Month, Bio14 – Precipitation of Driest Month, Bio15 – Precipitation Seasonality, Bio16 – Precipitation of Wettest Quarter, Bio17 – Precipitation of Driest Quarter, Bio18 – Precipitation of Warmest Quarter, Bio19 – Precipitation of Coldest Quarter.

See Figure S1 for key to Indian states.


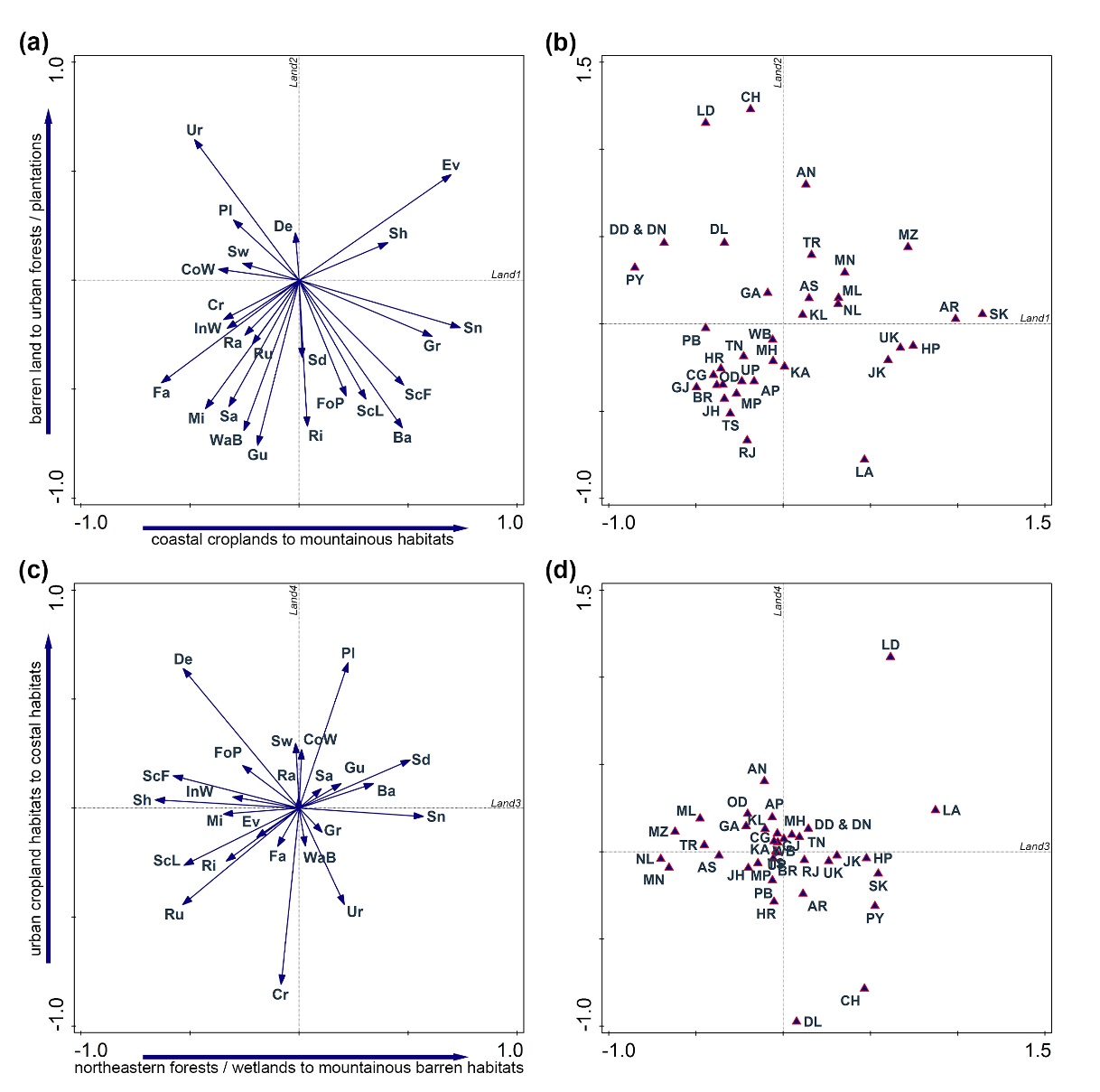


FIGURE S3. Unconstrained ordination of land covers variables

PCA biplots showing the mutual positions of 24 land covers variables at the first and the second ordination axes (a), positions of Indian federal states along the first and second axes (b), positions of the variables at the third and fourth ordination axes (c), and positions of Indian federal states along the third and fourth axes (d).

Key, land covers variables (from Bhuvan 2023): Cr – Crop land, Sh – Current Shifting cultivation, Fa – Fallow, Pl – Plantation, Ba – Barren rocky, Gu – Gullied / Ravinous, Ra – Rann, Sa – Salt affected land, Sd – Sandy area, ScL – Scrub land, Mi – Mining, Ru – Rural, Ur – Urban, De – Deciduous, Ev – Evergreen / Semi Evergreen, FoP – Forest Plantation, ScF – Scrub Forest, Sw – Swamp / Mangrove, Gr – Grass / Grazing land, Sn – Snow, InW – Inland wetland, CoW – Coastal wetland, Ri – River / Stream / Canals, WaB – Waterbodies.

See Figure S1 for key to Indian states.


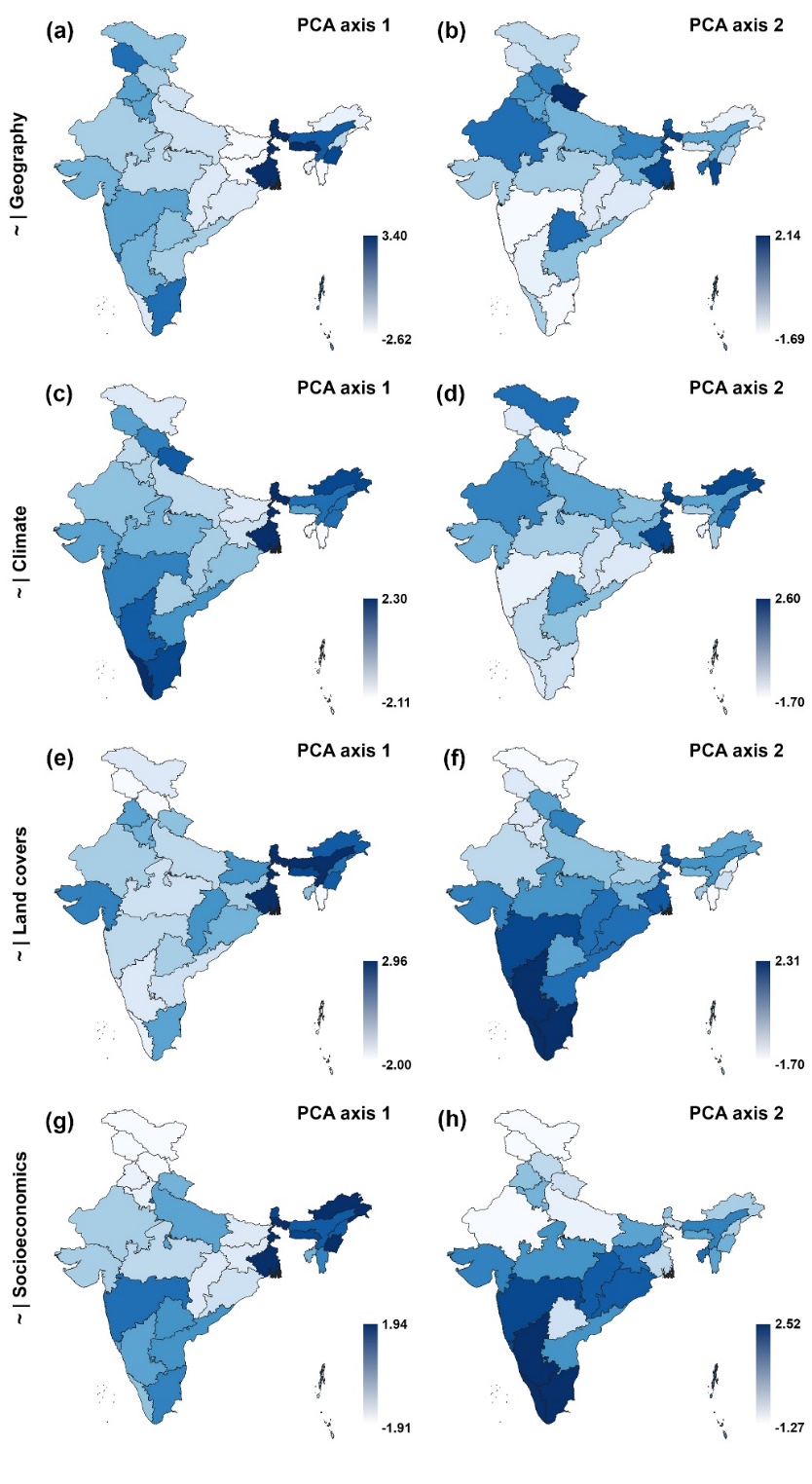


FIGURE S4. Visualisation of results of partial pPCA analyses, relating occurrences of 1386 butterflies recorded from the 36 federal states and union territories of the Republic of India, containing in covariables predictors describing geography (a, b), climate (c, d), land covers (e, f), and socioeconomics (g, h). Intensity of colours is proportional to values of loadings at the 1st to 2nd PCA axes of each predictor. See Table 2 for statistics of individual analyses.

TABLE S1. Details on preparation of phylogeny of Indian butterflies. All 1386 species recorded in India are listed, the column Taxon status differentiates species included in the phylogeny by Kawahara et al. 2023, and species added to the phylogeny by us.

| **Family** | **Species** | **Taxon status** |
| --- | --- | --- |
| Hesperiidae | *Badamia exclamationis* | Kawahara et al. 2023 |
| Hesperiidae | *Bibasis mahintha* | Added |
| Hesperiidae | *Bibasis sena* | Kawahara et al. 2023 |
| Hesperiidae | *Burara amara* | Kawahara et al. 2023 |
| Hesperiidae | *Burara anadi* | Added |
| Hesperiidae | *Burara etelka* | Added |
| Hesperiidae | *Burara gomata* | Added |
| Hesperiidae | *Burara harisa* | Kawahara et al. 2023 |
| Hesperiidae | *Burara jaina* | Added |
| Hesperiidae | *Burara oedipodea* | Kawahara et al. 2023 |
| Hesperiidae | *Burara vasutana* | Added |
| Hesperiidae | *Hasora anura* | Added |
| Hesperiidae | *Hasora badra* | Kawahara et al. 2023 |
| Hesperiidae | *Hasora chromus* | Kawahara et al. 2023 |
| Hesperiidae | *Hasora danda* | Added |
| Hesperiidae | *Hasora khoda* | Added |
| Hesperiidae | *Hasora leucospila* | Added |
| Hesperiidae | *Hasora salanga* | Added |
| Hesperiidae | *Hasora schoenherr* | Added |
| Hesperiidae | *Hasora taminatus* | Added |
| Hesperiidae | *Hasora vitta* | Added |
| Hesperiidae | *Choaspes benjaminii* | Kawahara et al. 2023 |
| Hesperiidae | *Choaspes furcatus* | Added |
| Hesperiidae | *Choaspes stigmatus* | Added |
| Hesperiidae | *Choaspes xanthopogon* | Added |
| Hesperiidae | *Lobocla liliana* | Kawahara et al. 2023 |
| Hesperiidae | *Capila jayadeva* | Kawahara et al. 2023 |
| Hesperiidae | *Capila lidderdali* | Added |
| Hesperiidae | *Capila pennicillatum* | Added |
| Hesperiidae | *Capila phanaeus* | Kawahara et al. 2023 |
| Hesperiidae | *Capila pieridoides* | Added |
| Hesperiidae | *Capila zennara* | Kawahara et al. 2023 |
| Hesperiidae | *Celaenorrhinus ambareesa* | Added |
| Hesperiidae | *Celaenorrhinus andamanicus* | Added |
| Hesperiidae | *Celaenorrhinus asmara* | Added |
| Hesperiidae | *Celaenorrhinus aspersa* | Added |
| Hesperiidae | *Celaenorrhinus aurivittatus* | Kawahara et al. 2023 |
| Hesperiidae | *Celaenorrhinus badius* | Added |
| Hesperiidae | *Celaenorrhinus dhanada* | Added |
| Hesperiidae | *Celaenorrhinus ficulnea* | Added |
| Hesperiidae | *Celaenorrhinus flavocincta* | Added |
| Hesperiidae | *Celaenorrhinus leucocera* | Kawahara et al. 2023 |
| Hesperiidae | *Celaenorrhinus morena* | Added |
| Hesperiidae | *Celaenorrhinus munda* | Added |
| Hesperiidae | *Celaenorrhinus nigricans* | Added |
| Hesperiidae | *Celaenorrhinus patula* | Kawahara et al. 2023 |
| Hesperiidae | *Celaenorrhinus pero* | Added |
| Hesperiidae | *Celaenorrhinus plagifera* | Added |
| Hesperiidae | *Celaenorrhinus pulomaya* | Added |
| Hesperiidae | *Celaenorrhinus putra* | Added |
| Hesperiidae | *Celaenorrhinus pyrrha* | Added |
| Hesperiidae | *Celaenorrhinus ratna* | Added |
| Hesperiidae | *Celaenorrhinus ruficornis* | Added |
| Hesperiidae | *Celaenorrhinus sumitra* | Added |
| Hesperiidae | *Celaenorrhinus tibetanus* | Added |
| Hesperiidae | *Celaenorrhinus zea* | Added |
| Hesperiidae | *Tapena thwaitesi* | Kawahara et al. 2023 |
| Hesperiidae | *Darpa hanria* | Added |
| Hesperiidae | *Darpa pteria* | Kawahara et al. 2023 |
| Hesperiidae | *Darpa striata* | Added |
| Hesperiidae | *Odina decoratus* | Added |
| Hesperiidae | *Coladenia agni* | Added |
| Hesperiidae | *Coladenia agnioides* | Added |
| Hesperiidae | *Coladenia hoenei* | Added |
| Hesperiidae | *Coladenia indrani* | Added |
| Hesperiidae | *Coladenia laxmi* | Added |
| Hesperiidae | *Satarupa gopala* | Kawahara et al. 2023 |
| Hesperiidae | *Satarupa splendens* | Added |
| Hesperiidae | *Satarupa zulla* | Added |
| Hesperiidae | *Seseria dohertyi* | Kawahara et al. 2023 |
| Hesperiidae | *Seseria sambara* | Added |
| Hesperiidae | *Pintara pinwilli* | Kawahara et al. 2023 |
| Hesperiidae | *Pintara tabrica* | Added |
| Hesperiidae | *Chamunda chamunda* | Kawahara et al. 2023 |
| Hesperiidae | *Gerosis bhagava* | Kawahara et al. 2023 |
| Hesperiidae | *Gerosis phisara* | Added |
| Hesperiidae | *Gerosis sinica* | Added |
| Hesperiidae | *Tagiades calligana* | Added |
| Hesperiidae | *Tagiades cohaerens* | Added |
| Hesperiidae | *Tagiades gana* | Kawahara et al. 2023 |
| Hesperiidae | *Tagiades japetus* | Kawahara et al. 2023 |
| Hesperiidae | *Tagiades menaka* | Added |
| Hesperiidae | *Tagiades parra* | Added |
| Hesperiidae | *Tagiades litigiosa* | Kawahara et al. 2023 |
| Hesperiidae | *Mooreana trichoneura* | Added |
| Hesperiidae | *Ctenoptilum multiguttata* | Added |
| Hesperiidae | *Ctenoptilum vasava* | Kawahara et al. 2023 |
| Hesperiidae | *Odontoptilum angulata* | Kawahara et al. 2023 |
| Hesperiidae | *Caprona agama* | Added |
| Hesperiidae | *Caprona alida* | Added |
| Hesperiidae | *Caprona ransonnetii* | Added |
| Hesperiidae | *Pseudocoladenia dan* | Kawahara et al. 2023 |
| Hesperiidae | *Pseudocoladenia fabia* | Added |
| Hesperiidae | *Pseudocoladenia fatih* | Added |
| Hesperiidae | *Pseudocoladenia fatua* | Added |
| Hesperiidae | *Pseudocoladenia festa* | Kawahara et al. 2023 |
| Hesperiidae | *Sarangesa purendra* | Added |
| Hesperiidae | *Sarangesa dasahara* | Kawahara et al. 2023 |
| Hesperiidae | *Gomalia elma* | Kawahara et al. 2023 |
| Hesperiidae | *Carcharodus alceae* | Kawahara et al. 2023 |
| Hesperiidae | *Carcharodus dravira* | Added |
| Hesperiidae | *Spialia carnea* | Added |
| Hesperiidae | *Spialia doris* | Added |
| Hesperiidae | *Spialia galba* | Added |
| Hesperiidae | *Spialia rubicunda* | Added |
| Hesperiidae | *Spialia zebra* | Added |
| Hesperiidae | *Spialia orbifer** | Kawahara et al. 2023 |
| Hesperiidae | *Erynnis pelias* | Added |
| Hesperiidae | *Erynnis pathan* | Added |
| Hesperiidae | *Pyrgus alpinus* | Added |
| Hesperiidae | *Pyrgus cashmirensis* | Added |
| Hesperiidae | *Pyrgus darwazica* | Added |
| Hesperiidae | *Carterocephalus avanti* | Added |
| Hesperiidae | *Baracus vittatus* | Kawahara et al. 2023 |
| Hesperiidae | *Ampittia subvittatus* | Kawahara et al. 2023 |
| Hesperiidae | *Ampittia dioscorides* | Kawahara et al. 2023 |
| Hesperiidae | *Ampittia maroides* | Added |
| Hesperiidae | *Aeromachus dubius* | Added |
| Hesperiidae | *Aeromachus jhora* | Added |
| Hesperiidae | *Aeromachus kali* | Added |
| Hesperiidae | *Aeromachus pygmaeus* | Added |
| Hesperiidae | *Aeromachus stigmatus* | Kawahara et al. 2023 |
| Hesperiidae | *Sebastonyma dolopia* | Kawahara et al. 2023 |
| Hesperiidae | *Sovia grahami* | Added |
| Hesperiidae | *Sovia lucasii* | Added |
| Hesperiidae | *Sovia malta* | Added |
| Hesperiidae | *Sovia separata* | Added |
| Hesperiidae | *Halpemorpha hyrtacus* | Added |
| Hesperiidae | *Pedesta masuriensis* | Added |
| Hesperiidae | *Pedesta panda* | Added |
| Hesperiidae | *Pedesta pandita* | Added |
| Hesperiidae | *Thoressa aina* | Added |
| Hesperiidae | *Thoressa astigmata* | Added |
| Hesperiidae | *Thoressa cerata* | Added |
| Hesperiidae | *Thoressa evershedi* | Added |
| Hesperiidae | *Thoressa fusca* | Added |
| Hesperiidae | *Thoressa gupta* | Added |
| Hesperiidae | *Thoressa honorei* | Added |
| Hesperiidae | *Thoressa hyrie* | Added |
| Hesperiidae | *Thoressa masoni* | Kawahara et al. 2023 |
| Hesperiidae | *Thoressa sitala* | Added |
| Hesperiidae | *Halpe arcuata* | Added |
| Hesperiidae | *Halpe filda* | Added |
| Hesperiidae | *Halpe flava* | Added |
| Hesperiidae | *Halpe hauxwelli* | Added |
| Hesperiidae | *Halpe homolea* | Added |
| Hesperiidae | *Halpe knyvetti* | Added |
| Hesperiidae | *Halpe kumara* | Added |
| Hesperiidae | *Halpe kusala* | Added |
| Hesperiidae | *Halpe porus* | Kawahara et al. 2023 |
| Hesperiidae | *Halpe sikkima* | Added |
| Hesperiidae | *Halpe wantona* | Added |
| Hesperiidae | *Halpe zema* | Added |
| Hesperiidae | *Halpe zola* | Added |
| Hesperiidae | *Pithauria marsena* | Added |
| Hesperiidae | *Pithauria murdava* | Added |
| Hesperiidae | *Pithauria stramineipennis* | Kawahara et al. 2023 |
| Hesperiidae | *Apostictopterus fuliginosus* | Kawahara et al. 2023 |
| Hesperiidae | *Astictopterus jama* | Kawahara et al. 2023 |
| Hesperiidae | *Actinor radians* | Added |
| Hesperiidae | *Iambrix salsala* | Kawahara et al. 2023 |
| Hesperiidae | *Koruthaialos butleri* | Kawahara et al. 2023 |
| Hesperiidae | *Koruthaialos rubecula* | Kawahara et al. 2023 |
| Hesperiidae | *Koruthaialos sindu* | Added |
| Hesperiidae | *Psolos fuligo* | Kawahara et al. 2023 |
| Hesperiidae | *Stimula swinhoei* | Kawahara et al. 2023 |
| Hesperiidae | *Ancistroides nigrita* | Added |
| Hesperiidae | *Notocrypta curvifascia* | Kawahara et al. 2023 |
| Hesperiidae | *Notocrypta feisthamelii* | Kawahara et al. 2023 |
| Hesperiidae | *Notocrypta paralysos* | Added |
| Hesperiidae | *Udaspes folus* | Kawahara et al. 2023 |
| Hesperiidae | *Arnetta atkinsoni* | Added |
| Hesperiidae | *Arnetta mercara* | Added |
| Hesperiidae | *Arnetta vindhiana* | Added |
| Hesperiidae | *Scobura cephala* | Kawahara et al. 2023 |
| Hesperiidae | *Scobura cephaloides* | Added |
| Hesperiidae | *Scobura isota* | Kawahara et al. 2023 |
| Hesperiidae | *Scobura phiditia* | Added |
| Hesperiidae | *Scobura tytleri* | Added |
| Hesperiidae | *Scobura parawoolletti* | Added |
| Hesperiidae | *Suada swerga* | Kawahara et al. 2023 |
| Hesperiidae | *Suastus gremius* | Added |
| Hesperiidae | *Suastus minutus* | Kawahara et al. 2023 |
| Hesperiidae | *Cupitha purreea* | Kawahara et al. 2023 |
| Hesperiidae | *Hyarotis adrastus* | Kawahara et al. 2023 |
| Hesperiidae | *Hyarotis microstictum* | Added |
| Hesperiidae | *Quedara basiflava* | Added |
| Hesperiidae | *Quedara monteithi* | Kawahara et al. 2023 |
| Hesperiidae | *Isma bonota* | Added |
| Hesperiidae | *Plastingia naga* | Kawahara et al. 2023 |
| Hesperiidae | *Salanoemia fuscicornis* | Added |
| Hesperiidae | *Salanoemia noemi* | Added |
| Hesperiidae | *Salanoemia sala* | Kawahara et al. 2023 |
| Hesperiidae | *Salanoemia tavoyana* | Added |
| Hesperiidae | *Pyroneura margherita* | Added |
| Hesperiidae | *Pyroneura niasana* | Added |
| Hesperiidae | *Lotongus sarala* | Added |
| Hesperiidae | *Zela zeus* | Kawahara et al. 2023 |
| Hesperiidae | *Gangara lebadea* | Kawahara et al. 2023 |
| Hesperiidae | *Gangara thyrsis* | Added |
| Hesperiidae | *Erionota acroleuca* | Kawahara et al. 2023 |
| Hesperiidae | *Erionota thrax* | Kawahara et al. 2023 |
| Hesperiidae | *Erionota torus* | Added |
| Hesperiidae | *Matapa aria* | Kawahara et al. 2023 |
| Hesperiidae | *Matapa cresta* | Added |
| Hesperiidae | *Matapa druna* | Added |
| Hesperiidae | *Matapa purpurascens* | Added |
| Hesperiidae | *Matapa sasivarna* | Added |
| Hesperiidae | *Creteus cyrina* | Kawahara et al. 2023 |
| Hesperiidae | *Pudicitia pholus* | Added |
| Hesperiidae | *Unkana ambasa* | Kawahara et al. 2023 |
| Hesperiidae | *Hidari bhawani* | Kawahara et al. 2023 |
| Hesperiidae | *Pirdana hyela* | Kawahara et al. 2023 |
| Hesperiidae | *Pirdana distanti* | Added |
| Hesperiidae | *Eogenes lesliei* | Added |
| Hesperiidae | *Eogenes alcides* | Kawahara et al. 2023 |
| Hesperiidae | *Zographetus ogygia* | Kawahara et al. 2023 |
| Hesperiidae | *Zographetus rama* | Added |
| Hesperiidae | *Zographetus satwa* | Added |
| Hesperiidae | *Zographetus dzonguensis* | Added |
| Hesperiidae | *Hesperia comma* | Kawahara et al. 2023 |
| Hesperiidae | *Ochlodes brahma* | Added |
| Hesperiidae | *Ochlodes siva* | Added |
| Hesperiidae | *Ochlodes subhyalina* | Added |
| Hesperiidae | *Gegenes nostrodamus* | Added |
| Hesperiidae | *Gegenes pumilio* | Kawahara et al. 2023 |
| Hesperiidae | *Parnara ganga* | Added |
| Hesperiidae | *Parnara guttatus* | Kawahara et al. 2023 |
| Hesperiidae | *Parnara bada* | Added |
| Hesperiidae | *Borbo bevani* | Added |
| Hesperiidae | *Borbo cinnara* | Kawahara et al. 2023 |
| Hesperiidae | *Pelopidas agna* | Added |
| Hesperiidae | *Pelopidas assamensis* | Added |
| Hesperiidae | *Pelopidas conjuncta* | Added |
| Hesperiidae | *Pelopidas mathias* | Kawahara et al. 2023 |
| Hesperiidae | *Pelopidas sinensis* | Added |
| Hesperiidae | *Pelopidas subochracea* | Added |
| Hesperiidae | *Pelopidas thrax* | Added |
| Hesperiidae | *Polytremis discreta* | Added |
| Hesperiidae | *Polytremis eltola* | Added |
| Hesperiidae | *Polytremis lubricans* | Kawahara et al. 2023 |
| Hesperiidae | *Polytremis minuta* | Added |
| Hesperiidae | *Baoris chapmani* | Added |
| Hesperiidae | *Baoris farri* | Kawahara et al. 2023 |
| Hesperiidae | *Baoris pagana* | Added |
| Hesperiidae | *Baoris unicolor* | Added |
| Hesperiidae | *Caltoris aurociliata* | Added |
| Hesperiidae | *Caltoris bromus* | Added |
| Hesperiidae | *Caltoris brunnea* | Added |
| Hesperiidae | *Caltoris cahira* | Added |
| Hesperiidae | *Caltoris canaraica* | Added |
| Hesperiidae | *Caltoris confusa* | Added |
| Hesperiidae | *Caltoris cormasa* | Kawahara et al. 2023 |
| Hesperiidae | *Caltoris kumara* | Added |
| Hesperiidae | *Caltoris philippina* | Added |
| Hesperiidae | *Caltoris plebeia* | Added |
| Hesperiidae | *Caltoris sirius* | Kawahara et al. 2023 |
| Hesperiidae | *Caltoris tulsi* | Added |
| Hesperiidae | *Iton semamora* | Kawahara et al. 2023 |
| Hesperiidae | *Taractrocera ceramas* | Added |
| Hesperiidae | *Taractrocera danna* | Added |
| Hesperiidae | *Taractrocera maevius* | Kawahara et al. 2023 |
| Hesperiidae | *Oriens concinna* | Added |
| Hesperiidae | *Oriens gola* | Kawahara et al. 2023 |
| Hesperiidae | *Oriens goloides* | Added |
| Hesperiidae | *Oriens paragola* | Added |
| Hesperiidae | *Potanthus confucius* | Added |
| Hesperiidae | *Potanthus dara* | Added |
| Hesperiidae | *Potanthus ganda* | Added |
| Hesperiidae | *Potanthus flavus* | Added |
| Hesperiidae | *Potanthus hetaerus* | Added |
| Hesperiidae | *Potanthus juno* | Added |
| Hesperiidae | *Potanthus lydia* | Added |
| Hesperiidae | *Potanthus mara* | Added |
| Hesperiidae | *Potanthus mingo* | Added |
| Hesperiidae | *Potanthus nesta* | Added |
| Hesperiidae | *Potanthus pallidus* | Added |
| Hesperiidae | *Potanthus palnia* | Added |
| Hesperiidae | *Potanthus pava* | Added |
| Hesperiidae | *Potanthus pseudomaesa* | Added |
| Hesperiidae | *Potanthus rectifasciata* | Added |
| Hesperiidae | *Potanthus sita* | Added |
| Hesperiidae | *Potanthus trachala* | Added |
| Hesperiidae | *Telicota augias* | Kawahara et al. 2023 |
| Hesperiidae | *Telicota bambusae* | Added |
| Hesperiidae | *Telicota besta* | Added |
| Hesperiidae | *Telicota colon* | Added |
| Hesperiidae | *Telicota linna* | Added |
| Hesperiidae | *Telicota ohara* | Added |
| Hesperiidae | *Cephrenes acalle* | Added |
| Papilionidae | *Losaria coon* | Kawahara et al. 2023 |
| Papilionidae | *Losaria rhodifer* | Added |
| Papilionidae | *Pachliopta aristolochiae* | Kawahara et al. 2023 |
| Papilionidae | *Pachliopta hector* | Added |
| Papilionidae | *Pachliopta pandiyana* | Added |
| Papilionidae | *Troides aeacus* | Added |
| Papilionidae | *Troides helena* | Added |
| Papilionidae | *Troides minos* | Added |
| Papilionidae | *Atrophaneura aidoneus* | Added |
| Papilionidae | *Atrophaneura varuna* | Added |
| Papilionidae | *Atrophaneura sycorax* | Added |
| Papilionidae | *Byasa crassipes* | Added |
| Papilionidae | *Byasa dasarada* | Added |
| Papilionidae | *Byasa latreillei* | Added |
| Papilionidae | *Byasa nevilli* | Added |
| Papilionidae | *Byasa plutonius* | Added |
| Papilionidae | *Byasa polla* | Added |
| Papilionidae | *Byasa polyeuctes* | Added |
| Papilionidae | *Papilio agenor* | Added |
| Papilionidae | *Papilio agestor* | Added |
| Papilionidae | *Papilio alcmenor* | Added |
| Papilionidae | *Papilio arcturus* | Added |
| Papilionidae | *Papilio bianor* | Added |
| Papilionidae | *Papilio bootes* | Added |
| Papilionidae | *Papilio buddha* | Added |
| Papilionidae | *Papilio castor* | Added |
| Papilionidae | *Papilio chaon* | Added |
| Papilionidae | *Papilio clytia* | Kawahara et al. 2023 |
| Papilionidae | *Papilio crino* | Added |
| Papilionidae | *Papilio daksha* | Added |
| Papilionidae | *Papilio demoleus* | Added |
| Papilionidae | *Papilio dravidarum* | Added |
| Papilionidae | *Papilio elephenor* | Added |
| Papilionidae | *Papilio epycides* | Added |
| Papilionidae | *Papilio helenus* | Added |
| Papilionidae | *Papilio janaka* | Added |
| Papilionidae | *Papilio krishna* | Added |
| Papilionidae | *Papilio liomedon* | Added |
| Papilionidae | *Papilio machaon* | Kawahara et al. 2023 |
| Papilionidae | *Papilio memnon* | Kawahara et al. 2023 |
| Papilionidae | *Papilio noblei* | Added |
| Papilionidae | *Papilio palinurus* | Added |
| Papilionidae | *Papilio paradoxa* | Added |
| Papilionidae | *Papilio paris* | Added |
| Papilionidae | *Papilio polyctor* | Added |
| Papilionidae | *Papilio polytes* | Kawahara et al. 2023 |
| Papilionidae | *Papilio prexaspes* | Added |
| Papilionidae | *Papilio protenor* | Kawahara et al. 2023 |
| Papilionidae | *Papilio slateri* | Kawahara et al. 2023 |
| Papilionidae | *Papilio xuthus* | Kawahara et al. 2023 |
| Papilionidae | *Graphium adonarensis* | Added |
| Papilionidae | *Graphium agamemnon* | Kawahara et al. 2023 |
| Papilionidae | *Graphium agetes* | Added |
| Papilionidae | *Graphium albociliatis* | Added |
| Papilionidae | *Graphium antiphates* | Added |
| Papilionidae | *Graphium aristeus* | Added |
| Papilionidae | *Graphium arycles* | Added |
| Papilionidae | *Graphium chironides* | Added |
| Papilionidae | *Graphium cloanthus* | Added |
| Papilionidae | *Graphium doson* | Added |
| Papilionidae | *Graphium epaminondas* | Added |
| Papilionidae | *Graphium eurous* | Added |
| Papilionidae | *Graphium eurypylus* | Added |
| Papilionidae | *Graphium macareus* | Added |
| Papilionidae | *Graphium garhwalica* | Added |
| Papilionidae | *Graphium paphus* | Added |
| Papilionidae | *Graphium megarus* | Added |
| Papilionidae | *Graphium nomius* | Added |
| Papilionidae | *Graphium septentrionicolus* | Added |
| Papilionidae | *Graphium sarpedon* | Kawahara et al. 2023 |
| Papilionidae | *Graphium teredon* | Added |
| Papilionidae | *Graphium xenocles* | Added |
| Papilionidae | *Lamproptera curius* | Kawahara et al. 2023 |
| Papilionidae | *Lamproptera meges* | Kawahara et al. 2023 |
| Papilionidae | *Meandrusa lachinus* | Kawahara et al. 2023 |
| Papilionidae | *Meandrusa payeni* | Added |
| Papilionidae | *Teinopalpus imperialis* | Kawahara et al. 2023 |
| Papilionidae | *Bhutanitis lidderdalii* | Added |
| Papilionidae | *Bhutanitis ludlowi* | Added |
| Papilionidae | *Parnassius actius* | Added |
| Papilionidae | *Parnassius epaphus* | Added |
| Papilionidae | *Parnassius jacquemontii* | Added |
| Papilionidae | *Parnassius tianschanicus* | Added |
| Papilionidae | *Parnassius charltonius* | Added |
| Papilionidae | *Parnassius loxias* | Added |
| Papilionidae | *Parnassius augustus* | Added |
| Papilionidae | *Parnassius acdestis* | Added |
| Papilionidae | *Parnassius staudingeri* | Added |
| Papilionidae | *Parnassius stenosemus* | Added |
| Papilionidae | *Parnassius stoliczkanus* | Added |
| Papilionidae | *Parnassius acco* | Added |
| Papilionidae | *Parnassius maharaja* | Added |
| Papilionidae | *Parnassius hardwickii* | Added |
| Papilionidae | *Parnassius simo* | Added |
| Papilionidae | *Parnassius hunnyngtoni* | Added |
| Papilionidae | *Parnassius dongalaica* | Added |
| Pieridae | *Catopsilia pomona* | Added |
| Pieridae | *Catopsilia pyranthe* | Added |
| Pieridae | *Catopsilia scylla* | Added |
| Pieridae | *Dercas verhuelli* | Kawahara et al. 2023 |
| Pieridae | *Dercas lycorias* | Kawahara et al. 2023 |
| Pieridae | *Gonepteryx amintha* | Added |
| Pieridae | *Gonepteryx chitralensis* | Added |
| Pieridae | *Gonepteryx mahaguru* | Added |
| Pieridae | *Gonepteryx nepalensis* | Added |
| Pieridae | *Gandaca harina* | Kawahara et al. 2023 |
| Pieridae | *Eurema andersonii* | Added |
| Pieridae | *Eurema blanda* | Added |
| Pieridae | *Eurema drona (=Eurema brigitta)* | Added |
| Pieridae | *Eurema hecabe* | Added |
| Pieridae | *Eurema laeta* | Added |
| Pieridae | *Eurema nilgiriensis* | Added |
| Pieridae | *Eurema simulatrix* | Added |
| Pieridae | *Colias alpherakii* | Added |
| Pieridae | *Colias berylla* | Added |
| Pieridae | *Colias cocandica* | Added |
| Pieridae | *Colias dubia* | Added |
| Pieridae | *Colias eogene* | Added |
| Pieridae | *Colias erate* | Added |
| Pieridae | *Colias fieldii* | Added |
| Pieridae | *Colias ladakensis* | Added |
| Pieridae | *Colias leechi* | Added |
| Pieridae | *Colias marcopolo* | Added |
| Pieridae | *Colias nilagiriensis* | Added |
| Pieridae | *Colias nina* | Added |
| Pieridae | *Colias stoliczkana* | Added |
| Pieridae | *Colias thrasibulus* | Added |
| Pieridae | *Colias wiskotti* | Added |
| Pieridae | *Leptosia nina* | Added |
| Pieridae | *Baltia butleri* | Kawahara et al. 2023 |
| Pieridae | *Baltia shawii* | Kawahara et al. 2023 |
| Pieridae | *Baltia sikkima* | Added |
| Pieridae | *Mesapia peloria* | Added |
| Pieridae | *Aporia agathon* | Kawahara et al. 2023 |
| Pieridae | *Aporia harrietae* | Added |
| Pieridae | *Aporia leucodice* | Added |
| Pieridae | *Aporia nabellica* | Added |
| Pieridae | *Aporia soracta* | Added |
| Pieridae | *Pieris ajaka* | Added |
| Pieridae | *Pieris brassicae* | Kawahara et al. 2023 |
| Pieridae | *Pieris canidia* | Added |
| Pieridae | *Pieris deota* | Added |
| Pieridae | *Pieris dubernardi* | Added |
| Pieridae | *Pieris extensa* | Added |
| Pieridae | *Pieris krueperi* | Added |
| Pieridae | *Pieris melaina* | Added |
| Pieridae | *Pieris erutae* | Added |
| Pieridae | *Pieris tadokoroi* | Added |
| Pieridae | *Pieris rapae* | Kawahara et al. 2023 |
| Pieridae | *Pieris naganum* | Added |
| Pieridae | *Pontia callidice* | Added |
| Pieridae | *Pontia chloridice* | Added |
| Pieridae | *Pontia daplidice* | Kawahara et al. 2023 |
| Pieridae | *Pontia glauconome* | Added |
| Pieridae | *Pontia sherpae** | Added |
| Pieridae | *Appias albina* | Added |
| Pieridae | *Appias cardena** | Added |
| Pieridae | *Appias indra* | Added |
| Pieridae | *Appias lalage* | Added |
| Pieridae | *Appias libythea* | Kawahara et al. 2023 |
| Pieridae | *Appias lyncida* | Added |
| Pieridae | *Appias galba* | Added |
| Pieridae | *Appias olferna* | Added |
| Pieridae | *Appias paulina* | Kawahara et al. 2023 |
| Pieridae | *Appias wardii* | Added |
| Pieridae | *Saletara liberia* | Kawahara et al. 2023 |
| Pieridae | *Prioneris philonome* | Added |
| Pieridae | *Prioneris sita* | Added |
| Pieridae | *Prioneris thestylis* | Kawahara et al. 2023 |
| Pieridae | *Belenois aurota* | Added |
| Pieridae | *Cepora nadina* | Added |
| Pieridae | *Cepora nerissa* | Kawahara et al. 2023 |
| Pieridae | *Delias acalis* | Added |
| Pieridae | *Delias agostina* | Added |
| Pieridae | *Delias belladonna* | Added |
| Pieridae | *Delias berinda* | Added |
| Pieridae | *Delias descombesi* | Kawahara et al. 2023 |
| Pieridae | *Delias eucharis* | Added |
| Pieridae | *Delias hyparete* | Added |
| Pieridae | *Delias lativitta* | Added |
| Pieridae | *Delias sanaca* | Added |
| Pieridae | *Delias pasithoe* | Kawahara et al. 2023 |
| Pieridae | *Euchloe daphalis* | Added |
| Pieridae | *Ixias marianne* | Added |
| Pieridae | *Ixias pyrene* | Kawahara et al. 2023 |
| Pieridae | *Colotis amata* | Kawahara et al. 2023 |
| Pieridae | *Colotis aurora* | Added |
| Pieridae | *Colotis danae* | Added |
| Pieridae | *Colotis etrida* | Added |
| Pieridae | *Colotis fausta* | Added |
| Pieridae | *Colotis phisadia* | Added |
| Pieridae | *Colotis protractus* | Added |
| Pieridae | *Pareronia avatar* | Added |
| Pieridae | *Pareronia ceylanica* | Added |
| Pieridae | *Pareronia hippia* | Added |
| Pieridae | *Hebomoia glaucippe* | Kawahara et al. 2023 |
| Riodinidae | *Zemeros flegyas* | Kawahara et al. 2023 |
| Riodinidae | *Dodona adonira* | Added |
| Riodinidae | *Dodona dipoea* | Added |
| Riodinidae | *Dodona deodata* | Kawahara et al. 2023 |
| Riodinidae | *Dodona durga* | Added |
| Riodinidae | *Dodona egeon* | Added |
| Riodinidae | *Dodona eugenes* | Added |
| Riodinidae | *Dodona longicaudata* | Added |
| Riodinidae | *Dodona ouida* | Added |
| Riodinidae | *Polycaena tamerlana* | Kawahara et al. 2023 |
| Riodinidae | *Stiboges nymphidia* | Added |
| Riodinidae | *Abisara abnormis* | Added |
| Riodinidae | *Abisara attenuata* | Added |
| Riodinidae | *Abisara bifasciata* | Kawahara et al. 2023 |
| Riodinidae | *Abisara burnii* | Kawahara et al. 2023 |
| Riodinidae | *Abisara chela* | Added |
| Riodinidae | *Abisara echerius* | Added |
| Riodinidae | *Abisara fylla* | Added |
| Riodinidae | *Abisara neophron* | Added |
| Riodinidae | *Abisara saturata* | Added |
| Riodinidae | *Taxila haquinus* | Kawahara et al. 2023 |
| Lycaenidae | *Curetis acuta* | Added |
| Lycaenidae | *Curetis bulis* | Kawahara et al. 2023 |
| Lycaenidae | *Curetis naga* | Added |
| Lycaenidae | *Curetis saronis* | Kawahara et al. 2023 |
| Lycaenidae | *Curetis siva* | Added |
| Lycaenidae | *Curetis thetis* | Added |
| Lycaenidae | *Poritia erycinoides* | Added |
| Lycaenidae | *Poritia hewitsoni* | Kawahara et al. 2023 |
| Lycaenidae | *Simiskina phalena* | Added |
| Lycaenidae | *Liphyra brassolis* | Kawahara et al. 2023 |
| Lycaenidae | *Allotinus drumila* | Added |
| Lycaenidae | *Allotinus subviolaceus* | Added |
| Lycaenidae | *Allotinus unicolor* | Kawahara et al. 2023 |
| Lycaenidae | *Allotinus taras** | Added |
| Lycaenidae | *Logania distanti* | Added |
| Lycaenidae | *Logania marmorata** | Added |
| Lycaenidae | *Logania watsoniana* | Added |
| Lycaenidae | *Miletus chinensis* | Added |
| Lycaenidae | *Miletus mallus* | Added |
| Lycaenidae | *Miletus nymphis* | Added |
| Lycaenidae | *Taraka hamada* | Kawahara et al. 2023 |
| Lycaenidae | *Spalgis baiongus* | Added |
| Lycaenidae | *Spalgis epius* | Kawahara et al. 2023 |
| Lycaenidae | *Lycaena aditya* | Added |
| Lycaenidae | *Lycaena alpherakii* | Added |
| Lycaenidae | *Lycaena evansii* | Added |
| Lycaenidae | *Lycaena kasyapa* | Added |
| Lycaenidae | *Lycaena panava* | Added |
| Lycaenidae | *Lycaena phlaeas* | Added |
| Lycaenidae | *Heliophorus androcles* | Added |
| Lycaenidae | *Heliophorus bakeri* | Added |
| Lycaenidae | *Heliophorus brahma* | Added |
| Lycaenidae | *Heliophorus epicles* | Kawahara et al. 2023 |
| Lycaenidae | *Heliophorus hybrida* | Added |
| Lycaenidae | *Heliophorus ila* | Added |
| Lycaenidae | *Heliophorus indicus* | Added |
| Lycaenidae | *Heliophorus kohimensis* | Added |
| Lycaenidae | *Heliophorus moorei* | Added |
| Lycaenidae | *Heliophorus oda* | Added |
| Lycaenidae | *Heliophorus sena* | Added |
| Lycaenidae | *Heliophorus tamu* | Added |
| Lycaenidae | *Apharitis acamas* | Added |
| Lycaenidae | *Apharitis chitralensis* | Added |
| Lycaenidae | *Apharitis lilacinus* | Added |
| Lycaenidae | *Spindasis abnormis* | Added |
| Lycaenidae | *Spindasis conjuncta** | Added |
| Lycaenidae | *Spindasis elima* | Added |
| Lycaenidae | *Spindasis elwesi* | Added |
| Lycaenidae | *Spindasis evansii* | Added |
| Lycaenidae | *Spindasis ictis* | Added |
| Lycaenidae | *Spindasis lohita* | Added |
| Lycaenidae | *Spindasis meghamalaiensis* | Added |
| Lycaenidae | *Spindasis mishmisensis* | Added |
| Lycaenidae | *Spindasis nipalicus* | Added |
| Lycaenidae | *Spindasis rukma* | Added |
| Lycaenidae | *Spindasis rukmini* | Added |
| Lycaenidae | *Spindasis schistacea* | Added |
| Lycaenidae | *Spindasis syama* | Added |
| Lycaenidae | *Spindasis vulcanus* | Added |
| Lycaenidae | *Spindasis zhengweilie* | Added |
| Lycaenidae | *Chaetoprocta baileyi* | Added |
| Lycaenidae | *Chaetoprocta odata* | Kawahara et al. 2023 |
| Lycaenidae | *Euaspa milionia* | Kawahara et al. 2023 |
| Lycaenidae | *Euaspa miyashitai* | Added |
| Lycaenidae | *Euaspa mikamii* | Added |
| Lycaenidae | *Euaspa motokii* | Added |
| Lycaenidae | *Euaspa pavo* | Added |
| Lycaenidae | *Shizuyaozephyrus ziha* | Added |
| Lycaenidae | *Fujiokaozephyrus tsangkie* | Added |
| Lycaenidae | *Iwaseozephyrus mandara* | Added |
| Lycaenidae | *Esakiozephyrus icana* | Added |
| Lycaenidae | *Yamamotozephyrus kwangtungensis* | Kawahara et al. 2023 |
| Lycaenidae | *Chrysozephyrus disparatus* | Added |
| Lycaenidae | *Chrysozephyrus duma* | Added |
| Lycaenidae | *Chrysozephyrus dumoides* | Added |
| Lycaenidae | *Chrysozephyrus intermedius* | Added |
| Lycaenidae | *Chrysozephyrus kabrua* | Added |
| Lycaenidae | *Chrysozephyrus sandersi* | Added |
| Lycaenidae | *Chrysozephyrus sikkimensis* | Added |
| Lycaenidae | *Chrysozephyrus tytleri* | Added |
| Lycaenidae | *Chrysozephyrus vittatus* | Added |
| Lycaenidae | *Chrysozephyrus zoa* | Added |
| Lycaenidae | *Neozephyrus suroia* | Added |
| Lycaenidae | *Shirozuozephyrus bhutanensis* | Added |
| Lycaenidae | *Shirozuozephyrus birupa* | Added |
| Lycaenidae | *Shirozuozephyrus jakamensis* | Added |
| Lycaenidae | *Shirozuozephyrus khasia* | Added |
| Lycaenidae | *Shirozuozephyrus kirbariensis* | Added |
| Lycaenidae | *Shirozuozephyrus paona* | Added |
| Lycaenidae | *Shirozuozephyrus triloka* | Added |
| Lycaenidae | *Inomataozephyrus assamicus* | Added |
| Lycaenidae | *Inomataozephyrus syla* | Added |
| Lycaenidae | *Thermozephyrus ataxus* | Added |
| Lycaenidae | *Leucantigius atayalicus* | Kawahara et al. 2023 |
| Lycaenidae | *Amblopala avidiena* | Kawahara et al. 2023 |
| Lycaenidae | *Arhopala aberrans* | Added |
| Lycaenidae | *Arhopala abseus* | Added |
| Lycaenidae | *Arhopala ace* | Added |
| Lycaenidae | *Arhopala aeeta* | Added |
| Lycaenidae | *Arhopala agrata* | Added |
| Lycaenidae | *Arhopala alea* | Added |
| Lycaenidae | *Arhopala allata* | Added |
| Lycaenidae | *Arhopala alax* | Added |
| Lycaenidae | *Arhopala alesia* | Added |
| Lycaenidae | *Arhopala amantes* | Added |
| Lycaenidae | *Arhopala ammonides* | Added |
| Lycaenidae | *Arhopala anarte* | Added |
| Lycaenidae | *Arhopala anthelus* | Added |
| Lycaenidae | *Arhopala ariel* | Added |
| Lycaenidae | *Arhopala arvina* | Added |
| Lycaenidae | *Arhopala asinarus* | Added |
| Lycaenidae | *Arhopala asopia* | Added |
| Lycaenidae | *Arhopala athada* | Added |
| Lycaenidae | *Arhopala atrax* | Added |
| Lycaenidae | *Arhopala aurelia* | Added |
| Lycaenidae | *Arhopala bazaloides* | Added |
| Lycaenidae | *Arhopala bazalus* | Kawahara et al. 2023 |
| Lycaenidae | *Arhopala belphoebe* | Added |
| Lycaenidae | *Arhopala birmana* | Added |
| Lycaenidae | *Arhopala camdeo* | Added |
| Lycaenidae | *Arhopala centaurus* | Added |
| Lycaenidae | *Arhopala comica* | Added |
| Lycaenidae | *Arhopala curiosa* | Added |
| Lycaenidae | *Arhopala democritus* | Added |
| Lycaenidae | *Arhopala dispar* | Added |
| Lycaenidae | *Arhopala dodonaea* | Added |
| Lycaenidae | *Arhopala eumolphus* | Added |
| Lycaenidae | *Arhopala fulla* | Added |
| Lycaenidae | *Arhopala ganesa* | Added |
| Lycaenidae | *Arhopala hellenore* | Added |
| Lycaenidae | *Arhopala khamti* | Added |
| Lycaenidae | *Arhopala nicevillei* | Added |
| Lycaenidae | *Arhopala oenea* | Added |
| Lycaenidae | *Arhopala opalina** | Added |
| Lycaenidae | *Arhopala paraganesa* | Added |
| Lycaenidae | *Arhopala paramuta* | Added |
| Lycaenidae | *Arhopala paralea* | Added |
| Lycaenidae | *Arhopala perimuta* | Added |
| Lycaenidae | *Arhopala rama* | Added |
| Lycaenidae | *Arhopala selta* | Added |
| Lycaenidae | *Arhopala silhetensis* | Added |
| Lycaenidae | *Arhopala singla* | Added |
| Lycaenidae | *Arhopala zeta* | Added |
| Lycaenidae | *Thaduka multicaudata* | Kawahara et al. 2023 |
| Lycaenidae | *Apporasa atkinsoni* | Kawahara et al. 2023 |
| Lycaenidae | *Mahathala ameria* | Kawahara et al. 2023 |
| Lycaenidae | *Flos anniella* | Added |
| Lycaenidae | *Flos apidanus* | Kawahara et al. 2023 |
| Lycaenidae | *Flos adriana* | Added |
| Lycaenidae | *Flos areste* | Added |
| Lycaenidae | *Flos asoka* | Added |
| Lycaenidae | *Flos chinensis* | Added |
| Lycaenidae | *Flos diardi* | Added |
| Lycaenidae | *Flos fulgida* | Added |
| Lycaenidae | *Mota massyla* | Kawahara et al. 2023 |
| Lycaenidae | *Surendra quercetorum* | Kawahara et al. 2023 |
| Lycaenidae | *Zinaspa todara* | Added |
| Lycaenidae | *Zesius chrysomallus* | Kawahara et al. 2023 |
| Lycaenidae | *Amblypodia anita* | Added |
| Lycaenidae | *Iraota rochana* | Kawahara et al. 2023 |
| Lycaenidae | *Iraota timoleon* | Added |
| Lycaenidae | *Catapaecilma major* | Kawahara et al. 2023 |
| Lycaenidae | *Catapaecilma subochrea* | Added |
| Lycaenidae | *Acupicta delicatum* | Added |
| Lycaenidae | *Loxura atymnus* | Kawahara et al. 2023 |
| Lycaenidae | *Yasoda tripunctata* | Kawahara et al. 2023 |
| Lycaenidae | *Drina donina* | Kawahara et al. 2023 |
| Lycaenidae | *Horaga albimacula* | Added |
| Lycaenidae | *Horaga onyx* | Kawahara et al. 2023 |
| Lycaenidae | *Horaga syrinx* | Added |
| Lycaenidae | *Horaga viola* | Added |
| Lycaenidae | *Rathinda amor* | Kawahara et al. 2023 |
| Lycaenidae | *Cheritra freja* | Kawahara et al. 2023 |
| Lycaenidae | *Cheritrella truncipennis* | Kawahara et al. 2023 |
| Lycaenidae | *Ticherra acte* | Kawahara et al. 2023 |
| Lycaenidae | *Drupadia scaeva* | Added |
| Lycaenidae | *Pratapa deva* | Kawahara et al. 2023 |
| Lycaenidae | *Pratapa icetas* | Added |
| Lycaenidae | *Pratapa icetoides* | Added |
| Lycaenidae | *Tajuria albiplaga* | Added |
| Lycaenidae | *Tajuria cippus* | Kawahara et al. 2023 |
| Lycaenidae | *Tajuria culta* | Added |
| Lycaenidae | *Tajuria deudorix* | Added |
| Lycaenidae | *Tajuria diaeus* | Added |
| Lycaenidae | *Tajuria illurgioides* | Added |
| Lycaenidae | *Tajuria illurgis* | Added |
| Lycaenidae | *Tajuria isaeus* | Added |
| Lycaenidae | *Tajuria ister* | Added |
| Lycaenidae | *Tajuria jehana* | Added |
| Lycaenidae | *Tajuria luculenta* | Added |
| Lycaenidae | *Tajuria maculata* | Added |
| Lycaenidae | *Tajuria megistia* | Added |
| Lycaenidae | *Tajuria melastigma* | Added |
| Lycaenidae | *Tajuria yajna* | Added |
| Lycaenidae | *Dacalana cotys* | Added |
| Lycaenidae | *Dacalana penicilligera* | Added |
| Lycaenidae | *Maneca bhotea* | Kawahara et al. 2023 |
| Lycaenidae | *Creon cleobis* | Kawahara et al. 2023 |
| Lycaenidae | *Bullis buto* | Kawahara et al. 2023 |
| Lycaenidae | *Rachana jalindra* | Kawahara et al. 2023 |
| Lycaenidae | *Neocheritra fabronia* | Added |
| Lycaenidae | *Charana cepheis* | Added |
| Lycaenidae | *Charana mandarina* | Kawahara et al. 2023 |
| Lycaenidae | *Suasa lisides* | Kawahara et al. 2023 |
| Lycaenidae | *Britomartis cleoboides** | Kawahara et al. 2023 |
| Lycaenidae | *Remelana jangala* | Kawahara et al. 2023 |
| Lycaenidae | *Ancema blanka* | Added |
| Lycaenidae | *Ancema ctesia* | Kawahara et al. 2023 |
| Lycaenidae | *Hypolycaena erylus* | Added |
| Lycaenidae | *Hypolycaena narada* | Added |
| Lycaenidae | *Hypolycaena nilgirica* | Added |
| Lycaenidae | *Hypolycaena thecloides* | Added |
| Lycaenidae | *Chliaria kina* | Added |
| Lycaenidae | *Chliaria othona* | Added |
| Lycaenidae | *Zeltus amasa* | Added |
| Lycaenidae | *Deudorix epijarbas* | Kawahara et al. 2023 |
| Lycaenidae | *Deudorix gaetulia* | Added |
| Lycaenidae | *Virachola dohertyi* | Added |
| Lycaenidae | *Virachola isocrates* | Added |
| Lycaenidae | *Virachola kessuma* | Added |
| Lycaenidae | *Virachola perse* | Added |
| Lycaenidae | *Virachola similis* | Added |
| Lycaenidae | *Artipe eryx* | Kawahara et al. 2023 |
| Lycaenidae | *Sinthusa chandrana* | Added |
| Lycaenidae | *Sinthusa nasaka* | Added |
| Lycaenidae | *Sinthusa virgo* | Added |
| Lycaenidae | *Araotes lapithis* | Kawahara et al. 2023 |
| Lycaenidae | *Bindahara phocides* | Kawahara et al. 2023 |
| Lycaenidae | *Rapala damona* | Added |
| Lycaenidae | *Rapala dieneces* | Added |
| Lycaenidae | *Rapala extensa* | Added |
| Lycaenidae | *Rapala iarbus* | Added |
| Lycaenidae | *Rapala lankana* | Added |
| Lycaenidae | *Rapala manea* | Added |
| Lycaenidae | *Rapala melida* | Added |
| Lycaenidae | *Rapala nissa* | Added |
| Lycaenidae | *Rapala pheretima* | Added |
| Lycaenidae | *Rapala rectivitta* | Added |
| Lycaenidae | *Rapala refulgens* | Added |
| Lycaenidae | *Rapala rosacea* | Added |
| Lycaenidae | *Rapala rubida* | Added |
| Lycaenidae | *Rapala selira* | Added |
| Lycaenidae | *Rapala scintilla* | Added |
| Lycaenidae | *Rapala suffusa* | Added |
| Lycaenidae | *Rapala tara* | Added |
| Lycaenidae | *Rapala varuna* | Kawahara et al. 2023 |
| Lycaenidae | *Pamela dudgeonii* | Added |
| Lycaenidae | *Ahlbergia leechii* | Added |
| Lycaenidae | *Satyrium mackwoodi* | Added |
| Lycaenidae | *Satyrium deria* | Added |
| Lycaenidae | *Niphanda asialis* | Added |
| Lycaenidae | *Niphanda cymbia* | Added |
| Lycaenidae | *Anthene emolus* | Added |
| Lycaenidae | *Anthene lycaenina* | Added |
| Lycaenidae | *Una usta* | Kawahara et al. 2023 |
| Lycaenidae | *Orthomiella pontis* | Kawahara et al. 2023 |
| Lycaenidae | *Orthomiella rantaizana** | Added |
| Lycaenidae | *Petrelaea dana* | Kawahara et al. 2023 |
| Lycaenidae | *Nacaduba berenice* | Added |
| Lycaenidae | *Nacaduba beroe* | Added |
| Lycaenidae | *Nacaduba calauria* | Added |
| Lycaenidae | *Nacaduba hermus* | Added |
| Lycaenidae | *Nacaduba kurava* | Kawahara et al. 2023 |
| Lycaenidae | *Nacaduba pactolus* | Added |
| Lycaenidae | *Nacaduba pavana* | Added |
| Lycaenidae | *Nacaduba sanaya* | Added |
| Lycaenidae | *Nacaduba sinhala* | Added |
| Lycaenidae | *Nacaduba subperusia* | Added |
| Lycaenidae | *Prosotas aluta* | Added |
| Lycaenidae | *Prosotas bhutea* | Added |
| Lycaenidae | *Prosotas dubiosa* | Added |
| Lycaenidae | *Prosotas lutea* | Added |
| Lycaenidae | *Prosotas nora* | Kawahara et al. 2023 |
| Lycaenidae | *Prosotas noreia* | Added |
| Lycaenidae | *Prosotas pia* | Added |
| Lycaenidae | *Ionolyce helicon* | Kawahara et al. 2023 |
| Lycaenidae | *Catopyrops ancyra* | Kawahara et al. 2023 |
| Lycaenidae | *Caleta decidia* | Added |
| Lycaenidae | *Caleta elna* | Added |
| Lycaenidae | *Caleta roxus* | Added |
| Lycaenidae | *Discolampa ethion* | Kawahara et al. 2023 |
| Lycaenidae | *Jamides alecto* | Added |
| Lycaenidae | *Jamides bochus* | Kawahara et al. 2023 |
| Lycaenidae | *Jamides caeruleus* | Added |
| Lycaenidae | *Jamides celeno* | Added |
| Lycaenidae | *Jamides elpis* | Added |
| Lycaenidae | *Jamides ferrari* | Added |
| Lycaenidae | *Jamides kankena* | Added |
| Lycaenidae | *Jamides pura* | Added |
| Lycaenidae | *Catochrysops panormus* | Added |
| Lycaenidae | *Catochrysops strabo* | Kawahara et al. 2023 |
| Lycaenidae | *Lampides boeticus* | Kawahara et al. 2023 |
| Lycaenidae | *Leptotes plinius* | Added |
| Lycaenidae | *Castalius rosimon* | Kawahara et al. 2023 |
| Lycaenidae | *Tarucus ananda* | Added |
| Lycaenidae | *Tarucus balkanicus* | Added |
| Lycaenidae | *Tarucus callinara* | Added |
| Lycaenidae | *Tarucus hazara* | Added |
| Lycaenidae | *Tarucus indicus* | Added |
| Lycaenidae | *Tarucus nara* | Added |
| Lycaenidae | *Tarucus venosus* | Added |
| Lycaenidae | *Tarucus waterstradti* | Added |
| Lycaenidae | *Zizeeria karsandra* | Kawahara et al. 2023 |
| Lycaenidae | *Pseudozizeeria maha* | Kawahara et al. 2023 |
| Lycaenidae | *Zizina otis* | Kawahara et al. 2023 |
| Lycaenidae | *Zizula hylax* | Kawahara et al. 2023 |
| Lycaenidae | *Everes argiades* | Added |
| Lycaenidae | *Everes huegelii* | Added |
| Lycaenidae | *Everes lacturnus* | Added |
| Lycaenidae | *Cupido alainus* | Added |
| Lycaenidae | *Cupido buddhista** | Added |
| Lycaenidae | *Iolana gigantea* | Added |
| Lycaenidae | *Bothrinia chennelli* | Added |
| Lycaenidae | *Tongeia kala* | Added |
| Lycaenidae | *Tongeia pseudozuthus* | Added |
| Lycaenidae | *Shijimia moorei* | Added |
| Lycaenidae | *Talicada nyseus* | Kawahara et al. 2023 |
| Lycaenidae | *Pithecops fulgens* | Added |
| Lycaenidae | *Pithecops corvus* | Kawahara et al. 2023 |
| Lycaenidae | *Azanus jesous* | Added |
| Lycaenidae | *Azanus ubaldus* | Kawahara et al. 2023 |
| Lycaenidae | *Azanus uranus* | Added |
| Lycaenidae | *Neopithecops zalmora* | Kawahara et al. 2023 |
| Lycaenidae | *Megisba malaya* | Kawahara et al. 2023 |
| Lycaenidae | *Celastrina argiolus* | Kawahara et al. 2023 |
| Lycaenidae | *Celastrina gigas* | Added |
| Lycaenidae | *Celastrina hersilia* | Added |
| Lycaenidae | *Celastrina huegelii* | Added |
| Lycaenidae | *Celastrina lavendularis* | Added |
| Lycaenidae | *Celastrina oreas* | Added |
| Lycaenidae | *Lestranicus transpectus* | Kawahara et al. 2023 |
| Lycaenidae | *Celatoxia albidisca* | Added |
| Lycaenidae | *Celatoxia marginata* | Kawahara et al. 2023 |
| Lycaenidae | *Notarthrinus binghami* | Added |
| Lycaenidae | *Acytolepis lilacea* | Added |
| Lycaenidae | *Acytolepis puspa* | Kawahara et al. 2023 |
| Lycaenidae | *Oreolyce dohertyi* | Added |
| Lycaenidae | *Oreolyce vardhana* | Kawahara et al. 2023 |
| Lycaenidae | *Callenya melaena* | Kawahara et al. 2023 |
| Lycaenidae | *Monodontides musina* | Added |
| Lycaenidae | *Udara akasa* | Added |
| Lycaenidae | *Udara albocaerulea* | Kawahara et al. 2023 |
| Lycaenidae | *Udara dilecta* | Kawahara et al. 2023 |
| Lycaenidae | *Udara placidula* | Added |
| Lycaenidae | *Udara selma* | Added |
| Lycaenidae | *Udara singalensis* | Added |
| Lycaenidae | *Euchrysops cnejus* | Kawahara et al. 2023 |
| Lycaenidae | *Freyeria putli* | Added |
| Lycaenidae | *Freyeria trochylus* | Kawahara et al. 2023 |
| Lycaenidae | *Luthrodes pandava* | Added |
| Lycaenidae | *Chilades lajus* | Kawahara et al. 2023 |
| Lycaenidae | *Lachides parrhasius* | Added |
| Lycaenidae | *Turanana chitrali* | Added |
| Lycaenidae | *Pseudophilotes vicrama* | Added |
| Lycaenidae | *Phengaris atroguttata* | Kawahara et al. 2023 |
| Lycaenidae | *Plebejus eversmanni* | Added |
| Lycaenidae | *Plebejus samudra* | Added |
| Lycaenidae | *Aricia agestis* | Kawahara et al. 2023 |
| Lycaenidae | *Aricia artaxerxes* | Added |
| Lycaenidae | *Eumedonia astorica* | Added |
| Lycaenidae | *Eumedonia annulata* | Added |
| Lycaenidae | *Eumedonia eumedon* | Kawahara et al. 2023 |
| Lycaenidae | *Agriades arcaseia* | Added |
| Lycaenidae | *Agriades jaloka* | Added |
| Lycaenidae | *Agriades lehanus* | Added |
| Lycaenidae | *Agriades leela* | Added |
| Lycaenidae | *Agriades morsheadi** | Added |
| Lycaenidae | *Agriades sikhima* | Added |
| Lycaenidae | *Agriades pheretiades* | Added |
| Lycaenidae | *Agriades asiatica* | Added |
| Lycaenidae | *Pamiria chrysopis* | Kawahara et al. 2023 |
| Lycaenidae | *Pamiria galathea* | Added |
| Lycaenidae | *Pamiria metallica* | Added |
| Lycaenidae | *Pamiria omphisa* | Added |
| Lycaenidae | *Pamiria wojtusiaki* | Added |
| Lycaenidae | *Patricius younghusbandi* | Added |
| Lycaenidae | *Plebejidea loewii* | Kawahara et al. 2023 |
| Lycaenidae | *Kretania beani* | Added |
| Lycaenidae | *Kretania csomai* | Added |
| Lycaenidae | *Afarsia ashretha* | Added |
| Lycaenidae | *Afarsia hanna** | Added |
| Lycaenidae | *Alpherakya devanica* | Added |
| Lycaenidae | *Alpherakya bellona* | Added |
| Lycaenidae | *Alpherakya rupala* | Added |
| Lycaenidae | *Alpherakya sartoides* | Added |
| Lycaenidae | *Polyommatus ariana* | Added |
| Lycaenidae | *Polyommatus dux* | Added |
| Lycaenidae | *Polyommatus florenciae* | Added |
| Lycaenidae | *Polyommatus hunza* | Added |
| Lycaenidae | *Polyommatus icarus* | Kawahara et al. 2023 |
| Lycaenidae | *Polyommatus icadius* | Added |
| Lycaenidae | *Polyommatus pseuderos* | Added |
| Lycaenidae | *Polyommatus pulchellus* | Added |
| Lycaenidae | *Polyommatus stoliczkanus* | Added |
| Nymphalidae | *Danaus affinis* | Added |
| Nymphalidae | *Danaus chrysippus* | Kawahara et al. 2023 |
| Nymphalidae | *Danaus genutia* | Added |
| Nymphalidae | *Danaus melanippus* | Added |
| Nymphalidae | *Ideopsis juventa* | Added |
| Nymphalidae | *Ideopsis similis* | Added |
| Nymphalidae | *Parantica aglea* | Kawahara et al. 2023 |
| Nymphalidae | *Parantica agleoides* | Added |
| Nymphalidae | *Parantica melaneus* | Added |
| Nymphalidae | *Parantica swinhoei* | Added |
| Nymphalidae | *Parantica nilgiriensis* | Added |
| Nymphalidae | *Parantica pedonga* | Added |
| Nymphalidae | *Parantica sita* | Kawahara et al. 2023 |
| Nymphalidae | *Tirumala gautama* | Added |
| Nymphalidae | *Tirumala limniace* | Kawahara et al. 2023 |
| Nymphalidae | *Tirumala septentrionis* | Added |
| Nymphalidae | *Idea agamarschana* | Added |
| Nymphalidae | *Idea malabarica* | Added |
| Nymphalidae | *Euploea algea* | Added |
| Nymphalidae | *Euploea core* | Added |
| Nymphalidae | *Euploea crameri* | Added |
| Nymphalidae | *Euploea doubledayi* | Added |
| Nymphalidae | *Euploea eunice* | Added |
| Nymphalidae | *Euploea klugii* | Added |
| Nymphalidae | *Euploea midamus* | Added |
| Nymphalidae | *Euploea modesta* | Added |
| Nymphalidae | *Euploea mulciber* | Added |
| Nymphalidae | *Euploea phaenareta* | Kawahara et al. 2023 |
| Nymphalidae | *Euploea radamanthus* | Kawahara et al. 2023 |
| Nymphalidae | *Euploea sylvester* | Added |
| Nymphalidae | *Calinaga buddha* | Added |
| Nymphalidae | *Calinaga gautama* | Added |
| Nymphalidae | *Calinaga aborica* | Added |
| Nymphalidae | *Calinaga brahma* | Added |
| Nymphalidae | *Prothoe franck* | Kawahara et al. 2023 |
| Nymphalidae | *Polyura agraria* | Added |
| Nymphalidae | *Polyura arja* | Added |
| Nymphalidae | *Polyura bharata* | Added |
| Nymphalidae | *Polyura delphis* | Added |
| Nymphalidae | *Polyura dolon* | Added |
| Nymphalidae | *Polyura eudamippus* | Kawahara et al. 2023 |
| Nymphalidae | *Polyura moori* | Added |
| Nymphalidae | *Polyura narcaeus* | Added |
| Nymphalidae | *Polyura schreiber* | Added |
| Nymphalidae | *Charaxes aristogiton* | Added |
| Nymphalidae | *Charaxes bernardus* | Kawahara et al. 2023 |
| Nymphalidae | *Charaxes durnfordi* | Added |
| Nymphalidae | *Charaxes kahruba* | Added |
| Nymphalidae | *Charaxes marmax* | Added |
| Nymphalidae | *Charaxes psaphon* | Added |
| Nymphalidae | *Charaxes solon* | Added |
| Nymphalidae | *Faunis eumeus* | Kawahara et al. 2023 |
| Nymphalidae | *Faunis canens* | Added |
| Nymphalidae | *Aemona amathusia* | Kawahara et al. 2023 |
| Nymphalidae | *Stichophthalma camadeva* | Added |
| Nymphalidae | *Stichophthalma nourmahal* | Added |
| Nymphalidae | *Stichophthalma sparta* | Added |
| Nymphalidae | *Amathusia andamanensis* | Added |
| Nymphalidae | *Amathusia phidippus* | Kawahara et al. 2023 |
| Nymphalidae | *Amathuxidia amythaon* | Kawahara et al. 2023 |
| Nymphalidae | *Thaumantis diores* | Kawahara et al. 2023 |
| Nymphalidae | *Thauria lathyi* | Kawahara et al. 2023 |
| Nymphalidae | *Discophora deo* | Kawahara et al. 2023 |
| Nymphalidae | *Discophora lepida* | Added |
| Nymphalidae | *Discophora sondaica* | Added |
| Nymphalidae | *Discophora timora* | Added |
| Nymphalidae | *Enispe cycnus* | Added |
| Nymphalidae | *Enispe euthymius* | Kawahara et al. 2023 |
| Nymphalidae | *Enispe intermedia* | Added |
| Nymphalidae | *Elymnias cottonis* | Added |
| Nymphalidae | *Elymnias hypermnestra* | Kawahara et al. 2023 |
| Nymphalidae | *Elymnias malelas* | Added |
| Nymphalidae | *Elymnias nesaea* | Kawahara et al. 2023 |
| Nymphalidae | *Elymnias obnubila* | Added |
| Nymphalidae | *Elymnias panthera* | Added |
| Nymphalidae | *Elymnias patna* | Added |
| Nymphalidae | *Elymnias peali* | Added |
| Nymphalidae | *Elymnias penaga* | Added |
| Nymphalidae | *Elymnias vasudeva* | Added |
| Nymphalidae | *Neorina hilda* | Added |
| Nymphalidae | *Neorina patria* | Added |
| Nymphalidae | *Penthema lisarda* | Added |
| Nymphalidae | *Ethope himachala* | Added |
| Nymphalidae | *Melanitis leda* | Kawahara et al. 2023 |
| Nymphalidae | *Melanitis phedima* | Added |
| Nymphalidae | *Melanitis zitenius* | Added |
| Nymphalidae | *Cyllogenes janetae* | Added |
| Nymphalidae | *Cyllogenes suradeva* | Added |
| Nymphalidae | *Parantirrhoea marshalli* | Added |
| Nymphalidae | *Lethe andersoni* | Added |
| Nymphalidae | *Lethe atkinsonia* | Added |
| Nymphalidae | *Lethe baladeva* | Added |
| Nymphalidae | *Lethe bhairava* | Added |
| Nymphalidae | *Lethe brisanda* | Added |
| Nymphalidae | *Lethe chandica* | Added |
| Nymphalidae | *Lethe confusa* | Added |
| Nymphalidae | *Lethe dakwania* | Added |
| Nymphalidae | *Lethe dura* | Kawahara et al. 2023 |
| Nymphalidae | *Lethe distans* | Added |
| Nymphalidae | *Lethe drypetis* | Added |
| Nymphalidae | *Lethe elwesi* | Added |
| Nymphalidae | *Lethe europa* | Kawahara et al. 2023 |
| Nymphalidae | *Lethe goalpara* | Added |
| Nymphalidae | *Lethe gemina* | Added |
| Nymphalidae | *Lethe gulnihal* | Added |
| Nymphalidae | *Lethe hyrania (=Lethe isana)* | Added |
| Nymphalidae | *Lethe jalaurida* | Added |
| Nymphalidae | *Lethe kabrua* | Added |
| Nymphalidae | *Lethe kanjupkula* | Added |
| Nymphalidae | *Lethe kansa* | Added |
| Nymphalidae | *Lethe latiaris* | Added |
| Nymphalidae | *Lethe maitrya* | Added |
| Nymphalidae | *Lethe margaritae* | Added |
| Nymphalidae | *Lethe mekara* | Added |
| Nymphalidae | *Lethe moelleri* | Added |
| Nymphalidae | *Lethe naga* | Added |
| Nymphalidae | *Lethe nicetas* | Added |
| Nymphalidae | *Lethe nicetella* | Added |
| Nymphalidae | *Lethe ramadeva* | Added |
| Nymphalidae | *Lethe rohria* | Kawahara et al. 2023 |
| Nymphalidae | *Lethe satyavati* | Added |
| Nymphalidae | *Lethe scanda* | Added |
| Nymphalidae | *Lethe serbonis* | Added |
| Nymphalidae | *Lethe siderea* | Added |
| Nymphalidae | *Lethe sidonis* | Added |
| Nymphalidae | *Lethe sinorix* | Added |
| Nymphalidae | *Lethe sura* | Added |
| Nymphalidae | *Lethe tristigmata* | Added |
| Nymphalidae | *Lethe verma* | Added |
| Nymphalidae | *Lethe vindhya* | Added |
| Nymphalidae | *Lethe visrava* | Added |
| Nymphalidae | *Neope armandii* | Added |
| Nymphalidae | *Neope bhadra* | Kawahara et al. 2023 |
| Nymphalidae | *Neope pulaha* | Added |
| Nymphalidae | *Neope pulahina* | Added |
| Nymphalidae | *Neope pulahoides* | Added |
| Nymphalidae | *Neope yama* | Added |
| Nymphalidae | *Lasiommata maerula* | Added |
| Nymphalidae | *Lasiommata menava* | Added |
| Nymphalidae | *Lasiommata pakistana* | Added |
| Nymphalidae | *Lasiommata schakra* | Added |
| Nymphalidae | *Kirinia eversmanni* | Added |
| Nymphalidae | *Chonala masoni* | Added |
| Nymphalidae | *Rhaphicera moorei* | Added |
| Nymphalidae | *Rhaphicera satricus* | Added |
| Nymphalidae | *Orinoma damaris* | Kawahara et al. 2023 |
| Nymphalidae | *Heteropsis adolphei* | Added |
| Nymphalidae | *Mycalesis adamsonii* | Added |
| Nymphalidae | *Mycalesis anapita* | Added |
| Nymphalidae | *Mycalesis anaxias* | Added |
| Nymphalidae | *Mycalesis annamitica* | Added |
| Nymphalidae | *Mycalesis bethami* | Added |
| Nymphalidae | *Mycalesis davisoni* | Added |
| Nymphalidae | *Mycalesis evansii* | Added |
| Nymphalidae | *Mycalesis francisca* | Kawahara et al. 2023 |
| Nymphalidae | *Mycalesis gotama* | Added |
| Nymphalidae | *Mycalesis heri* | Added |
| Nymphalidae | *Mycalesis igilia* | Added |
| Nymphalidae | *Mycalesis intermedia* | Added |
| Nymphalidae | *Mycalesis lepcha* | Added |
| Nymphalidae | *Mycalesis malsara* | Added |
| Nymphalidae | *Mycalesis malsarida* | Added |
| Nymphalidae | *Mycalesis manii* | Added |
| Nymphalidae | *Mycalesis mestra* | Added |
| Nymphalidae | *Mycalesis mineus* | Added |
| Nymphalidae | *Mycalesis misenus* | Added |
| Nymphalidae | *Mycalesis mnasicles* | Kawahara et al. 2023 |
| Nymphalidae | *Mycalesis mystes* | Added |
| Nymphalidae | *Mycalesis nicotia* | Added |
| Nymphalidae | *Mycalesis oculus* | Added |
| Nymphalidae | *Mycalesis orcha* | Added |
| Nymphalidae | *Mycalesis orseis* | Added |
| Nymphalidae | *Mycalesis patiana** | Added |
| Nymphalidae | *Mycalesis patnia* | Added |
| Nymphalidae | *Mycalesis perseus* | Added |
| Nymphalidae | *Mycalesis radza* | Added |
| Nymphalidae | *Mycalesis suaveolens* | Added |
| Nymphalidae | *Mycalesis subdita* | Added |
| Nymphalidae | *Mycalesis visala* | Added |
| Nymphalidae | *Orsotriaena medus* | Kawahara et al. 2023 |
| Nymphalidae | *Zipaetis saitis* | Added |
| Nymphalidae | *Zipaetis scylax* | Added |
| Nymphalidae | *Erites falcipennis* | Added |
| Nymphalidae | *Coelites nothis* | Kawahara et al. 2023 |
| Nymphalidae | *Ragadia crisilda* | Added |
| Nymphalidae | *Hyponephele brevistigma* | Added |
| Nymphalidae | *Hyponephele carbonelli* | Added |
| Nymphalidae | *Hyponephele cheena* | Added |
| Nymphalidae | *Hyponephele chitralica* | Added |
| Nymphalidae | *Hyponephele coenonympha* | Added |
| Nymphalidae | *Hyponephele davendra* | Added |
| Nymphalidae | *Hyponephele tenuistigma* | Added |
| Nymphalidae | *Hyponephele pulchella* | Added |
| Nymphalidae | *Hyponephele pulchra* | Added |
| Nymphalidae | *Hyponephele hilaris* | Added |
| Nymphalidae | *Callerebia annada* | Kawahara et al. 2023 |
| Nymphalidae | *Callerebia baileyi* | Added |
| Nymphalidae | *Callerebia dibangensis* | Added |
| Nymphalidae | *Callerebia hybrida* | Added |
| Nymphalidae | *Callerebia nirmala* | Added |
| Nymphalidae | *Callerebia orixa* | Added |
| Nymphalidae | *Callerebia watsoni* | Added |
| Nymphalidae | *Callerebia scanda* | Added |
| Nymphalidae | *Callerebia suroia* | Added |
| Nymphalidae | *Paralasa chitralica* | Added |
| Nymphalidae | *Paralasa kalinda* | Kawahara et al. 2023 |
| Nymphalidae | *Paralasa mani* | Added |
| Nymphalidae | *Paralasa shallada* | Added |
| Nymphalidae | *Loxerebia narasingha* | Added |
| Nymphalidae | *Ypthima affectata* | Added |
| Nymphalidae | *Ypthima asterope* | Added |
| Nymphalidae | *Ypthima atra* | Added |
| Nymphalidae | *Ypthima baldus* | Added |
| Nymphalidae | *Ypthima bolanica** | Added |
| Nymphalidae | *Ypthima cantliei* | Added |
| Nymphalidae | *Ypthima ceylonica* | Added |
| Nymphalidae | *Ypthima chenu* | Added |
| Nymphalidae | *Ypthima davidsoni* | Added |
| Nymphalidae | *Ypthima dohertyi* | Added |
| Nymphalidae | *Ypthima fusca* | Added |
| Nymphalidae | *Ypthima hannyngtoni* | Added |
| Nymphalidae | *Ypthima huebneri* | Kawahara et al. 2023 |
| Nymphalidae | *Ypthima hyagriva* | Added |
| Nymphalidae | *Ypthima indecora* | Added |
| Nymphalidae | *Ypthima inica* | Added |
| Nymphalidae | *Ypthima kasmira* | Added |
| Nymphalidae | *Ypthima lisandra* | Added |
| Nymphalidae | *Ypthima lycus* | Added |
| Nymphalidae | *Ypthima methora* | Added |
| Nymphalidae | *Ypthima nareda* | Added |
| Nymphalidae | *Ypthima newara* | Added |
| Nymphalidae | *Ypthima nikaea* | Added |
| Nymphalidae | *Ypthima norma* | Added |
| Nymphalidae | *Ypthima parasakra* | Added |
| Nymphalidae | *Ypthima persimilis* | Added |
| Nymphalidae | *Ypthima philomela* | Added |
| Nymphalidae | *Ypthima sakra* | Added |
| Nymphalidae | *Ypthima savara* | Added |
| Nymphalidae | *Ypthima singala* | Added |
| Nymphalidae | *Ypthima sobrina** | Added |
| Nymphalidae | *Ypthima striata* | Added |
| Nymphalidae | *Ypthima watsoni* | Added |
| Nymphalidae | *Ypthima ypthimoides* | Added |
| Nymphalidae | *Oeneis buddha* | Added |
| Nymphalidae | *Paroeneis pumilus* | Added |
| Nymphalidae | *Paroeneis sikkimensis* | Added |
| Nymphalidae | *Karanasa astorica* | Added |
| Nymphalidae | *Karanasa bolorica* | Added |
| Nymphalidae | *Karanasa cadesia* | Added |
| Nymphalidae | *Karanasa huebneri* | Added |
| Nymphalidae | *Karanasa modesta* | Added |
| Nymphalidae | *Karanasa moorei* | Added |
| Nymphalidae | *Karanasa leechi* | Added |
| Nymphalidae | *Karanasa pupilata* | Added |
| Nymphalidae | *Karanasa rohtanga* | Added |
| Nymphalidae | *Satyrus alaica* | Added |
| Nymphalidae | *Satyrus pimpla* | Added |
| nymphalidae | *Aulocera brahminoides* | Added |
| Nymphalidae | *Aulocera brahminus* | Kawahara et al. 2023 |
| Nymphalidae | *Aulocera loha* | Kawahara et al. 2023 |
| Nymphalidae | *Aulocera padma* | Added |
| Nymphalidae | *Aulocera saraswati* | Added |
| Nymphalidae | *Aulocera swaha* | Added |
| Nymphalidae | *Hipparchia parisatis* | Added |
| Nymphalidae | *Chazara enervata* | Added |
| Nymphalidae | *Chazara heydenreichi* | Added |
| Nymphalidae | *Pseudochazara baldiva* | Added |
| Nymphalidae | *Pseudochazara droshica* | Added |
| Nymphalidae | *Pseudochazara lehana* | Added |
| Nymphalidae | *Kanetisa digna* | Added |
| Nymphalidae | *Neptis ananta* | Added |
| Nymphalidae | *Neptis armandia* | Added |
| Nymphalidae | *Neptis capnodes* | Added |
| Nymphalidae | *Neptis cartica* | Added |
| Nymphalidae | *Neptis clinia* | Added |
| Nymphalidae | *Neptis cydippe* | Added |
| Nymphalidae | *Neptis harita* | Added |
| Nymphalidae | *Neptis hylas* | Added |
| Nymphalidae | *Neptis ilira* | Added |
| Nymphalidae | *Neptis jumbah* | Added |
| Nymphalidae | *Neptis magadha* | Added |
| Nymphalidae | *Neptis mahendra* | Added |
| Nymphalidae | *Neptis manasa* | Added |
| Nymphalidae | *Neptis miah* | Added |
| Nymphalidae | *Neptis namba* | Added |
| Nymphalidae | *Neptis narayana* | Added |
| Nymphalidae | *Neptis nashona* | Added |
| Nymphalidae | *Neptis nata* | Added |
| Nymphalidae | *Neptis nemorum* | Added |
| Nymphalidae | *Neptis nycteus* | Added |
| Nymphalidae | *Neptis pseudovikasi* | Added |
| Nymphalidae | *Neptis radha* | Added |
| Nymphalidae | *Neptis sankara* | Added |
| Nymphalidae | *Neptis sappho* | Kawahara et al. 2023 |
| Nymphalidae | *Neptis soma* | Added |
| Nymphalidae | *Neptis zaida* | Added |
| Nymphalidae | *Phaedyma aspasia* | Added |
| Nymphalidae | *Phaedyma columella* | Kawahara et al. 2023 |
| Nymphalidae | *Lasippa tiga* | Added |
| Nymphalidae | *Lasippa monata* | Added |
| Nymphalidae | *Lasippa viraja* | Added |
| Nymphalidae | *Pantoporia assamica* | Added |
| Nymphalidae | *Pantoporia aurelia* | Added |
| Nymphalidae | *Pantoporia bieti* | Added |
| Nymphalidae | *Pantoporia hordonia* | Kawahara et al. 2023 |
| Nymphalidae | *Pantoporia paraka* | Added |
| Nymphalidae | *Pantoporia sandaka* | Added |
| Nymphalidae | *Lebadea martha* | Kawahara et al. 2023 |
| Nymphalidae | *Athyma asura* | Added |
| Nymphalidae | *Athyma cama* | Added |
| Nymphalidae | *Athyma nefte* | Added |
| Nymphalidae | *Athyma jina* | Added |
| Nymphalidae | *Athyma kanwa* | Added |
| Nymphalidae | *Athyma larymna* | Added |
| Nymphalidae | *Athyma opalina* | Added |
| Nymphalidae | *Athyma orientalis* | Added |
| Nymphalidae | *Athyma perius* | Kawahara et al. 2023 |
| Nymphalidae | *Athyma pravara* | Added |
| Nymphalidae | *Athyma punctata* | Added |
| Nymphalidae | *Athyma ranga* | Added |
| Nymphalidae | *Athyma reta* | Added |
| Nymphalidae | *Athyma rufula* | Added |
| Nymphalidae | *Athyma selenophora* | Added |
| Nymphalidae | *Athyma whitei* | Added |
| Nymphalidae | *Athyma zeroca* | Added |
| Nymphalidae | *Limenitis hydaspes* | Added |
| Nymphalidae | *Limenitis ligyes* | Added |
| Nymphalidae | *Limenitis rileyi* | Added |
| Nymphalidae | *Limenitis trivena* | Added |
| Nymphalidae | *Moduza procris* | Kawahara et al. 2023 |
| Nymphalidae | *Parasarpa dudu* | Added |
| Nymphalidae | *Parasarpa zayla* | Added |
| Nymphalidae | *Sumalia daraxa* | Kawahara et al. 2023 |
| Nymphalidae | *Sumalia zulema* | Kawahara et al. 2023 |
| Nymphalidae | *Auzakia danava* | Kawahara et al. 2023 |
| Nymphalidae | *Neurosigma siva* | Kawahara et al. 2023 |
| Nymphalidae | *Bhagadatta austenia* | Kawahara et al. 2023 |
| Nymphalidae | *Parthenos sylvia* | Kawahara et al. 2023 |
| Nymphalidae | *Abrota ganga* | Kawahara et al. 2023 |
| Nymphalidae | *Tanaecia cibaritis* | Added |
| Nymphalidae | *Tanaecia cocytus* | Added |
| Nymphalidae | *Tanaecia jahnu* | Added |
| Nymphalidae | *Tanaecia julii* | Added |
| Nymphalidae | *Tanaecia lepidea* | Kawahara et al. 2023 |
| Nymphalidae | *Dophla evelina* | Added |
| Nymphalidae | *Bassarona durga* | Added |
| Nymphalidae | *Bassarona iva* | Added |
| Nymphalidae | *Bassarona recta* | Added |
| Nymphalidae | *Bassarona teuta* | Kawahara et al. 2023 |
| Nymphalidae | *Euthalia aconthea* | Added |
| Nymphalidae | *Euthalia alpheda* | Added |
| Nymphalidae | *Euthalia anosia* | Added |
| Nymphalidae | *Euthalia confucius* | Added |
| Nymphalidae | *Euthalia curvifascia* | Added |
| Nymphalidae | *Euthalia duda* | Added |
| Nymphalidae | *Euthalia eriphylae* | Added |
| Nymphalidae | *Euthalia franciae* | Added |
| Nymphalidae | *Euthalia lengba* | Added |
| Nymphalidae | *Euthalia lubentina* | Kawahara et al. 2023 |
| Nymphalidae | *Euthalia malaccana* | Added |
| Nymphalidae | *Euthalia monina* | Added |
| Nymphalidae | *Euthalia nara* | Added |
| Nymphalidae | *Euthalia narayana* | Added |
| Nymphalidae | *Euthalia patala* | Added |
| Nymphalidae | *Euthalia phemius* | Added |
| Nymphalidae | *Euthalia sahadeva* | Added |
| Nymphalidae | *Euthalia saitaphernes* | Added |
| Nymphalidae | *Euthalia telchinia* | Added |
| Nymphalidae | *Euthalia thawgawa* | Added |
| Nymphalidae | *Symphaedra nais* | Kawahara et al. 2023 |
| Nymphalidae | *Lexias cyanipardus* | Added |
| Nymphalidae | *Lexias dirtea* | Added |
| Nymphalidae | *Lexias pardalis* | Added |
| Nymphalidae | *Argynnis aglaja* | Kawahara et al. 2023 |
| Nymphalidae | *Argynnis childreni* | Added |
| Nymphalidae | *Argynnis clara* | Added |
| Nymphalidae | *Argynnis hyperbius* | Kawahara et al. 2023 |
| Nymphalidae | *Argynnis jainadeva* | Added |
| Nymphalidae | *Argynnis kamala* | Added |
| Nymphalidae | *Argynnis laodice* | Added |
| Nymphalidae | *Argynnis pandora* | Added |
| Nymphalidae | *Argynnis westphali* | Added |
| Nymphalidae | *Issoria altissima* | Added |
| Nymphalidae | *Issoria gemmata* | Added |
| Nymphalidae | *Issoria issaea* | Added |
| Nymphalidae | *Issoria mackinnonii* | Added |
| Nymphalidae | *Clossiana erubescens* | Added |
| Nymphalidae | *Clossiana jerdoni* | Added |
| Nymphalidae | *Boloria generator* | Added |
| Nymphalidae | *Boloria pales* | Kawahara et al. 2023 |
| Nymphalidae | *Boloria sipora* | Added |
| Nymphalidae | *Phalanta alcippe* | Added |
| Nymphalidae | *Phalanta phalantha* | Kawahara et al. 2023 |
| Nymphalidae | *Cupha erymanthis* | Kawahara et al. 2023 |
| Nymphalidae | *Vagrans egista* | Kawahara et al. 2023 |
| Nymphalidae | *Vindula erota* | Added |
| Nymphalidae | *Algia fasciata* | Added |
| Nymphalidae | *Cirrochroa aoris* | Kawahara et al. 2023 |
| Nymphalidae | *Cirrochroa nicobarica* | Added |
| Nymphalidae | *Cirrochroa thais* | Added |
| Nymphalidae | *Cirrochroa tyche* | Added |
| Nymphalidae | *Acraea issoria* | Added |
| Nymphalidae | *Acraea terpsicore* | Added |
| Nymphalidae | *Cethosia biblis* | Kawahara et al. 2023 |
| Nymphalidae | *Cethosia cyane* | Added |
| Nymphalidae | *Cethosia nietneri* | Added |
| Nymphalidae | *Ariadne ariadne* | Kawahara et al. 2023 |
| Nymphalidae | *Ariadne merione* | Added |
| Nymphalidae | *Laringa horsfieldii* | Kawahara et al. 2023 |
| Nymphalidae | *Byblia ilithyia* | Kawahara et al. 2023 |
| Nymphalidae | *Rohana tonkiniana* | Added |
| Nymphalidae | *Rohana parisatis* | Kawahara et al. 2023 |
| Nymphalidae | *Rohana parvata* | Added |
| Nymphalidae | *Eulaceura manipurensis* | Added |
| Nymphalidae | *Chitoria naga* | Added |
| Nymphalidae | *Chitoria sordida* | Added |
| Nymphalidae | *Chitoria ulupi* | Added |
| Nymphalidae | *Mimathyma ambica* | Added |
| Nymphalidae | *Mimathyma bhavana* | Added |
| Nymphalidae | *Mimathyma chevana* | Kawahara et al. 2023 |
| Nymphalidae | *Dilipa morgiana* | Kawahara et al. 2023 |
| Nymphalidae | *Sephisa chandra* | Kawahara et al. 2023 |
| Nymphalidae | *Sephisa dichroa* | Added |
| Nymphalidae | *Helcyra hemina* | Kawahara et al. 2023 |
| Nymphalidae | *Herona marathus* | Kawahara et al. 2023 |
| Nymphalidae | *Euripus consimilis* | Added |
| Nymphalidae | *Euripus nyctelius* | Kawahara et al. 2023 |
| Nymphalidae | *Hestina nicevillei* | Added |
| Nymphalidae | *Hestina persimilis* | Kawahara et al. 2023 |
| Nymphalidae | *Hestinalis nama* | Kawahara et al. 2023 |
| Nymphalidae | *Sasakia funebris* | Added |
| Nymphalidae | *Cyrestis cocles* | Added |
| Nymphalidae | *Cyrestis tabula* | Added |
| Nymphalidae | *Cyrestis thyodamas* | Added |
| Nymphalidae | *Chersonesia intermedia* | Added |
| Nymphalidae | *Chersonesia risa* | Added |
| Nymphalidae | *Pseudergolis wedah* | Added |
| Nymphalidae | *Stibochiona nicea* | Added |
| Nymphalidae | *Dichorragia nesimachus* | Kawahara et al. 2023 |
| Nymphalidae | *Melitaea arcesia* | Added |
| Nymphalidae | *Melitaea balbina* | Added |
| Nymphalidae | *Melitaea chitralensis* | Added |
| Nymphalidae | *Melitaea fergana* | Added |
| Nymphalidae | *Melitaea nadezhdae* | Added |
| Nymphalidae | *Melitaea pallas* | Added |
| Nymphalidae | *Melitaea robertsi* | Added |
| Nymphalidae | *Melitaea shandura* | Added |
| Nymphalidae | *Symbrenthia brabira* | Added |
| Nymphalidae | *Symbrenthia doni* | Added |
| Nymphalidae | *Symbrenthia hypselis* | Added |
| Nymphalidae | *Symbrenthia lilaea* | Added |
| Nymphalidae | *Symbrenthia niphanda* | Added |
| Nymphalidae | *Symbrenthia silana* | Added |
| Nymphalidae | *Araschnia dohertyi* | Added |
| Nymphalidae | *Nymphalis antiopa* | Kawahara et al. 2023 |
| Nymphalidae | *Nymphalis l-album* | Added |
| Nymphalidae | *Nymphalis polychloros* | Added |
| Nymphalidae | *Nymphalis xanthomelas* | Kawahara et al. 2023 |
| Nymphalidae | *Aglais caschmirensis* | Added |
| Nymphalidae | *Aglais ladakensis* | Added |
| Nymphalidae | *Aglais rizana* | Added |
| Nymphalidae | *Kaniska canace* | Kawahara et al. 2023 |
| Nymphalidae | *Polygonia c-album* | Kawahara et al. 2023 |
| Nymphalidae | *Polygonia undina* | Added |
| Nymphalidae | *Vanessa cardui* | Added |
| Nymphalidae | *Vanessa indica* | Kawahara et al. 2023 |
| Nymphalidae | *Junonia almana* | Added |
| Nymphalidae | *Junonia atlites* | Added |
| Nymphalidae | *Junonia hierta* | Added |
| Nymphalidae | *Junonia iphita* | Added |
| Nymphalidae | *Junonia lemonias* | Added |
| Nymphalidae | *Junonia orithya* | Added |
| Nymphalidae | *Hypolimnas anomala* | Added |
| Nymphalidae | *Hypolimnas bolina* | Kawahara et al. 2023 |
| Nymphalidae | *Hypolimnas misippus* | Kawahara et al. 2023 |
| Nymphalidae | *Yoma sabina* | Kawahara et al. 2023 |
| Nymphalidae | *Kallima albofasciata* | Added |
| Nymphalidae | *Kallima horsfieldi* | Added |
| Nymphalidae | *Kallima inachus* | Added |
| Nymphalidae | *Kallima knyvetti* | Added |
| Nymphalidae | *Doleschallia bisaltide* | Kawahara et al. 2023 |
| Nymphalidae | *Rhinopalpa polynice* | Kawahara et al. 2023 |
| Nymphalidae | *Libythea lepita* | Kawahara et al. 2023 |
| Nymphalidae | *Libythea myrrha* | Added |
| Nymphalidae | *Libythea narina* | Added |
